# Sensitive asprosin detection in clinical samples reveals serum/saliva correlation and indicates cartilage as source for serum asprosin

**DOI:** 10.1038/s41598-022-05060-x

**Published:** 2022-01-25

**Authors:** Yousef A. T. Morcos, Steffen Lütke, Antje Tenbieg, Franz-Georg Hanisch, Galyna Pryymachuk, Nadin Piekarek, Thorben Hoffmann, Titus Keller, Ruth Janoschek, Anja Niehoff, Frank Zaucke, Jörg Dötsch, Eva Hucklenbruch-Rother, Gerhard Sengle

**Affiliations:** 1 Center for Biochemistry, Faculty of Medicine and University Hospital of Cologne, University of Cologne, Joseph-Stelzmann-Street 52, 50931 Cologne, Germany; 2 Department of Pediatrics and Adolescent Medicine, Faculty of Medicine and University Hospital Cologne, University of Cologne, Cologne, Germany; 3 Department of Anatomy I, Faculty of Medicine and University Hospital Cologne, University of Cologne, Cologne, Germany; 4 Institute of Biomechanics and Orthopaedics, German Sport University Cologne, Cologne, Germany; 5 Cologne Center for Musculoskeletal Biomechanics (CCMB), Faculty of Medicine and University Hospital of Cologne, University of Cologne, Cologne, Germany; 6 Dr. Rolf M. Schwiete Research Unit for Osteoarthritis, Department of Orthopaedics (Friedrichsheim), University Hospital, Goethe University, Frankfurt am Main, Germany; 7 Center for Molecular Medicine Cologne (CMMC), University of Cologne, Cologne, Germany

**Keywords:** Biochemistry, Biological techniques, Cell biology, Molecular biology, Biomarkers, Molecular medicine

## Abstract

The C-terminal pro-fibrillin-1 propeptide asprosin is described as white adipose tissue derived hormone that stimulates rapid hepatic glucose release and activates hunger-promoting hypothalamic neurons. Numerous studies proposed correlations of asprosin levels with clinical parameters. However, the enormous variability of reported serum and plasma asprosin levels illustrates the need for sensitive and reliable detection methods in clinical samples. Here we report on newly developed biochemical methods for asprosin concentration and detection in several body fluids including serum, plasma, saliva, breast milk, and urine. Since we found that glycosylation impacts human asprosin detection we analyzed its glycosylation profile. Employing a new sandwich ELISA revealed that serum and saliva asprosin correlate strongly, depend on biological sex, and feeding status. To investigate the contribution of connective tissue-derived asprosin to serum levels we screened two cohorts with described cartilage turnover. Serum asprosin correlated with COMP, a marker for cartilage degradation upon running exercise and after total hip replacement surgery. This together with our finding that asprosin is produced by primary human chondrocytes and expressed in human cartilage suggests a contribution of cartilage to serum asprosin. Furthermore, we determined asprosin levels in breast milk, and urine, for the first time, and propose saliva asprosin as an accessible clinical marker for future studies.

## Introduction

Recently, it was discovered that the C-terminal cleavage product of profibrillin-1 serves as a fasting-induced glucogenic protein hormone that modulates hepatic glucose release^1^. It is cleaved by the proprotein convertase furin in the secretory pathway and was termed “asprosin” after the Greek word for white, since it was first described to originate from white adipose tissue^1^. Asprosin is released by fibrillin-1 producing connective tissues, circulates in the blood, and is recruited to the liver, where it induces G protein-coupled activation of the cAMP-PKA pathway and stimulates rapid glucose release into the circulation^1^. Asprosin was also shown to directly activate appetite-promoting neurons of the hypothalamus^2^, and neutralization of circulating asprosin with a monoclonal antibody reduced appetite and body weight in obese mice and improved their glycemic profile^2^. Moreover, anti-asprosin monoclonal antibody therapy was recently shown to be promising in the treatment of metabolic syndrome as it reduced appetite and body weight in mouse models^3^.

Due to its proposed crucial metabolic function, several clinical studies have been conducted to establish a correlation of asprosin levels with obesity, diabetes mellitus type 1 and 2, insulin resistance, metabolic syndrome, and physical exercise^4–6^. However, reported measurements of asprosin in clinical samples showed considerable variations across studies raising serious concerns about the reliability of the applied ELISA detection approaches^5^. For instance, asprosin concentrations determined in blood samples from children showed huge variations of four orders of magnitude from < 1 ng/ml up to > 100 ng/ml^7–10^ (Supplementary Table 1). In adults reported asprosin concentrations in plasma and serum even range from < 0.5 ng to > 350 ng/ml (Supplementary Table 1). Additionally, the sensitivity of the employed ELISA kits appears to vary by more than one order of magnitude regarding standard detection range (0.156–10 ng/ml to 7.80–500 ng/ml), and sensitivity (0.1–2.1 ng/ml) (Supplementary Table 1).

This documented variability illustrates the need for new approaches for the sensitive and reliable measurement of asprosin in human body fluids. We therefore aimed to develop new biochemical tools and methods for asprosin detection in several body fluids. Since fibrillin-1 is known to be a structural component of the extracellular matrix architecture of connective tissue microenvironments^11^, it is plausible that connective tissue derived asprosin contributes to blood asprosin levels. To test a potential contribution of cartilage derived asprosin to total serum asprosin levels, we measured asprosin levels in two cohorts for which release of cartilage derived components into the blood were already documented by monitoring serum levels of cartilage oligomeric matrix protein (COMP), a marker for cartilage degradation and osteoarthritis^12,13^. We measured serum samples from athletes before and after mechanical loading by acute physical activity as well as and from osteoarthritis patients before and after hip replacement surgery. Our results not only provide new biochemical insight into the molecular requirements for sensitive asprosin detection in so far not investigated body fluids but also revealed a new correlation between asprosin levels and cartilage metabolism.

## Results

### Establishment of sensitive methods for human asprosin detection

#### Generation of recombinant human asprosin and analysis of its glycosylation profile

**Figure 1:**
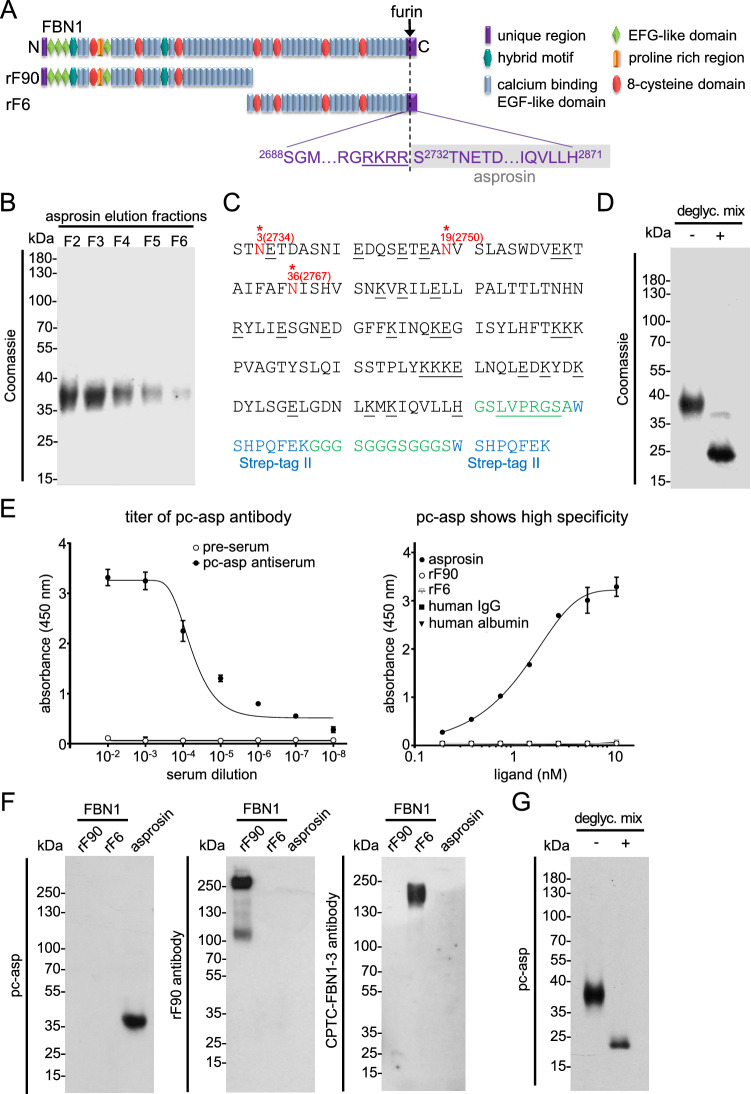
Generation of a specific polyclonal anti-asprosin antibody. (**A**) Domain structure of fibrillin-1 and its N- and C-terminal halves. The C-terminal propeptide sequence of fibrillin-1 after furin cleavage (furin cleavage site marked by arrow and dashed line, furin cleavage sequence underlined) yields asprosin (marked in grey). The 140 amino acids representing human asprosin S^2732^-H^2871^ were recombinantly produced in HEK293 cells. (**B**) Coomassie stained quality control gel of recombinantly produced and purified (> 95% purity) human asprosin. Asprosin was overexpressed with a C-terminally placed 2 × -Strep-tag II, and eluted fractions (F2-F6) after affinity chromatography were subjected to reducing SDS-PAGE using a 12% gel. (**C**) Mass spectrometry analysis revealed that all of the predicted three N-linked glycosylation sites (marked in red, position regarding the fibrillin-1 sequence in parentheses) are used as indicated by the + 1 mass shift expected for de-N-glycosylated peptides (see also Supplementary Table S2). Underlined residues mark predicted cleavage sites of V8 and trypsin used to generate peptides of de-N-glycosylated asprosin. No unequivocal assignments of O-glycosylation sites were possible on the basis of peptide masses in MS1 spectra. Residues representing linker regions are indicated in green, thrombin cleavage (LVPRGS) site is underlined, and Strep-tag II sequences are marked in blue. (**D**) Removal of N-linked, as well as many common O-linked glycans, after incubation in a deglycosylation mix results in significant loss of molecular weight of human asprosin. (**E**) (left) Polyclonal anti-asprosin serum (pc-asp anti-asprosin antibody) raised in rabbit showed a high titer in ELISA assay detecting coated asprosin (100 ng/well). Half maximum signal was already reached at a 1:10,000 dilution. Rabbit pre-serum (before rabbit immunization) served as a control. Recombinant asprosin was coated in duplicates (100 ng/well). (right) ELISA showing high specificity of the pc-asp antibody. No cross-reactivity to the N-terminal region of fibrillin-1 (rF90), C-terminal region of fibrillin-1 (rF6), human albumin, or human IgG was detected. Data points represent mean ± SD of duplicates. (**F**) (left) Pc-asp antibody specifically recognizes human asprosin in western blot analysis, with no observable cross-reactivity to the N-terminal region (rF90) and C-terminal region (rF6) of fibrillin-1. (middle) Detection of N-terminal fibrillin-1 (rF90) with labmade fibrillin-1 rF90 antibody. (right) Specific detection of the C-terminal fibrillin-1 (rF6) with CPTC-FBN1-3 antibody (DSHB, Iowa, USA) which was raised against a synthetic peptide in the rF6 region. (**G**) Reduced signal of deglycosylated asprosin in western blot by pc-asp anti-asprosin antibody. Data were analyzed using Graphpad Prism version 8.0.2.

For the generation of a specific polyclonal antiserum against asprosin, we recombinantly overexpressed the human asprosin protein sequence (Fig. 1C) in HEK293 EBNA cells with a C-terminally placed double Strep-tag II for efficient affinity purification. Quality control SDS-PAGE analysis showed that asprosin was obtained with high purity after resin-coupled streptactin affinity chromatography as assessed by Coomassie staining (Fig. 1B). The molecular weight of recombinant asprosin corresponded to approximately 37 kDa, suggesting that approximately 20 kDa were added by glycosylation in HEK293 cells when compared to its calculated mass based on the amino acid sequence (Fig. 1B,C). Deglycosylation by using an enzyme mixture to remove all N-linked as well as many common O-linked glycans resulted in a band at 20 kDa which corresponds to the calculated mass of the asprosin core protein (Fig. 1D,G).

It was previously predicted that human asprosin possesses three N-glycosylation sites^1^, and recently its N-glycosylation in yeast was investigated^14^. However, until now no data is available describing the actual sites and profiles of asprosin N-glycosylation used by human cells. To investigate these structural profiles of N-linked chains as well as to identify which N-glycosylation sites are used in human cells, we performed mass spectrometry of asprosin overexpressed in HEK293 cells after enzymatic glycan liberation and proteolytic digestion of de-N-glycosylated peptides. Profiles of PNGaseF liberated N-glycans were characterized by one dominant high-mannose-type chain (M5) and by a prominent series of core-fucosylated complex-type chains with bi- to tetra-antennarity and sialylation with up to four residues (Supplementary Fig. S1 and Table S2). Besides these major glycans with fully processed complex-type structures, a considerable proportion of incompletely processed species of the truncated -Gal series was detected. N-Glycosylation sites were identified in the peptide mass fingerprints after de-N-glycosylation and conversion of Asn to Asp by the + 1 mass shift (see Supplementary Fig. S3 and Table S3). According to these peptide mass fingerprints, all three predicted sites (N3 (N2734), N19 (N2750), and N36 (N2767)) were found to be glycosylated (Fig. 1C). Asprosin could be also O-glycosylated, as three sites in the amino-terminal region (Ser1, Thr5, Thr16) were predicted (NetOGlyc version 4.0.0.13). Chemical cleavage of O-linked chains by reductive beta-elimination revealed one signal in the MALDI survey spectrum at m/z 1706 that could be structurally assigned by PSD-MALDI-MS to a hexasaccharide alditol based on a core 2 tetrasaccharide substituted with two sialic acid residues (Supplementary Fig. S2). Based on peptide mass fingerprints of de-N-glycosylated asprosin peptides, no assignments of the O-glycosylation sites were possible.

#### Establishment of ELISA and SPR assays for sensitive asprosin detection

**Figure 2:**
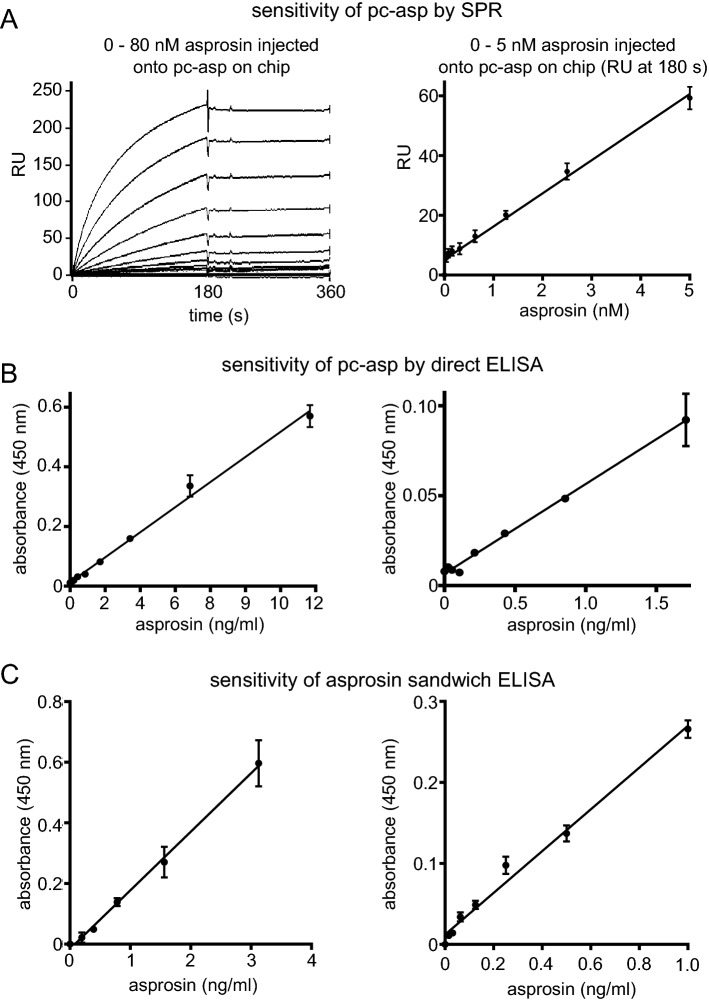
Establishment of assays for sensitive asprosin detection. (**A**) Testing of pc-asp sensitivity using surface plasmon resonance (SPR). (left) Sensorgrams showing detection of recombinant asprosin injected at 0–80 nM (0–2960 ng/ml) onto pc-asp immobilized to the sensor chip. (right) Robust and linear response of asprosin injected at 0–5 nM (0–185 ng/ml) onto pc-asp immobilized on chip. (**B)** Asprosin detection by direct ELISA using pc-asp as detector antibody (1:2000 dilution, 0.5 µg/ml). Robust and linear ELISA signal response of immobilized recombinant asprosin. (**C)** Sensitive detection of asprosin in solution by sandwich ELISA. Pc-asp was immobilized as capture antibody (3 μg/ml) and mab anti-asprosin (AdipoGen Life Sciences Inc., San Diego, USA) was used as detector antibody (1:2000 dilution, 0.5 µg/ml). Data points represent mean ± SD from duplicates. Data were analyzed using Graphpad Prism version 8.0.2.

Purified asprosin was injected into a rabbit for polyclonal antibody production. The obtained serum after 60 days of immunization showed a half-maximum titer at 1:10^4^ dilution. The affinity-purified antibody showed a high specificity against human asprosin and showed no cross-reactivity to mouse asprosin or the N-terminal and C-terminal halves of fibrillin-1 (Fig. 1A) when used in western blot and direct ELISA (Fig. 1E,F and Supplementary Fig. S5D). To assess the sensitivity of the newly generated polyclonal anti-asprosin antibody (pc-asp) we employed surface plasmon resonance (SPR). Anti-asprosin antibody was immobilized onto a sensor chip and a concentration series of recombinant asprosin (0–80 nM) was flown over in solution (Fig. 2A). Obtained signals showed a linear response still in the range of 0–1.25 nM (0–50 ng/ml) (Fig. 2A, (right)). An even higher sensitivity was observed by direct ELISA when asprosin was directly coated to the microtiter well, showing a linear response in the range between 0 and 1.0 ng/ml (0–28 pM) (Fig. 2B). Next, we established a sandwich ELISA by using the newly generated pc-asp as capture and a commercially available monoclonal anti-asprosin antibody (mab-asp) (clone Birdy-1, AdipoGen Life Sciences Inc.) as detector. Titration experiments showed a linear detection signal in the range of 0–0.15 ng/ml (0–4 pM) (Fig. 2C). The linear detection range of the asprosin sandwich ELISA was 0–50 ng/ml (Supplementary Fig. S4A). Coating concentration of pc-asp as capture antibody was optimized at 3 µg/ml, as higher coating concentrations lead to a marked decrease of the detection signal (Supplementary Fig. S4B). SPR measurements of immobilized asprosin showed that pc-asp has a molecular affinity in the < 1 nM range (K_D_ = 0.29 ± 0.30 nM), while the K_D_ of mab-asp is 75-fold higher (22 ± 2 nM) (Supplementary Fig. S5A). SPR experiments also revealed that the newly generated pc-asp has a more than 100-fold higher sensitivity for immobilized asprosin compared to mab-asp (Supplementary Fig. S5B) which was also indicated by direct ELISA measurements (Supplementary Fig. S5C). The established sandwich ELISA assay can detect human asprosin with a sensitivity of < 65 pg/ml with an inter-assay coefficient of variability (CV) < 6.6% (n = 32), and an intra-assay CV < 5.5% (n = 32). We also tested any potential cross-reactivity of pc-asp against mouse asprosin and human placensin, the C-terminal propeptide of fibrillin-2 which was recently proposed to also have a metabolic function^15^. In direct ELISA and western blot no signals of mouse asprosin were detectable (Supplementary Fig. S5D). Also our asprosin sandwich ELISA did not detect placensin which we also overexpressed und purified with a double C-terminal Strep-tag II (Supplementary Fig. S5E–G). This finding even excluded a potential cross-reactivity of pc-asp against the Strep-tag II sequence. Deglycosylation resulted in a better detection sensitivity of asprosin by mab in ELISA (Supplementary Fig. S5H (right)) and western blot (Supplementary Fig. S5I), while direct ELISA detection by pc-asp was not altered (Supplementary Fig. S5H (left)).

#### Affinity column chromatography allows specific asprosin concentration and determination in serum and urine

**Figure 3:**
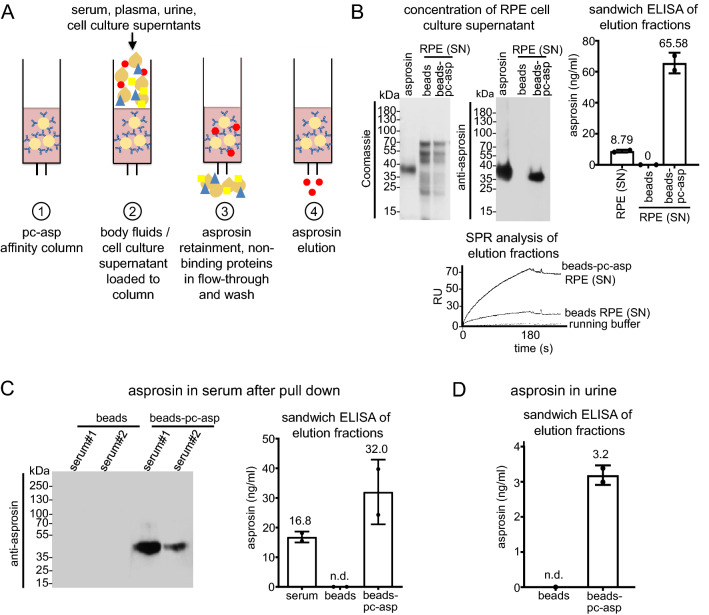
Affinity column chromatography allows specific asprosin concentration from serum and urine. (**A**) Schematic diagram showing the pull-down approach using pc-asp antibody for asprosin enrichment in cell culture supernatant and human biological samples. (**B**) (top left) Coomassie stain of 10% SDS-PAGE gel of recombinantly expressed and affinity-purified asprosin, pull-down from RPE media supernatant with CNBr beads (control), and pc-asp antibody coupled to CNBr beads. (top middle) Western Blot with mab anti-asprosin (1:1000, 1 µg/ml) showing specific bands of the recombinantly expressed asprosin and the endogenous asprosin of the RPE supernatant (1 L serum-free cell culture supernatant) pull-down from pc-asp antibody coupled to CNBr beads. (top right) Detection of asprosin in RPE cell culture media before and after pull-down enrichment showing an eight-fold increase of asprosin concentration by sandwich ELISA. (Bottom) Sensorgram of SPR showing detection of endogenous asprosin in RPE supernatant pull-down from CNBr beads coupled to the pc-asp antibody. Data obtained from two independent pull-down experiments. (**C**) (left) Western Blot analysis with mab anti-asprosin (1:1000 dilution, 1 µg/ml) of the pull-down from two human serum samples with pc-asp antibody coupled to CNBr beads showing endogenous asprosin band. (right) Quantification of the endogenous asprosin levels using sandwich ELISA in the human serum sample (1 ml serum diluted 1:10 with 10 mM Tris–HCl, pH 7.5) before and after enrichment of asprosin concentration by asprosin pull-down approach. (**D**) Detection of asprosin in human urine (200 ml) after enrichment using the pull-down approach. Data obtained from urine sample subjected to two independent pull- down experiments. Data were analyzed using Graphpad Prism version 8.0.2.

Depending on the sensitivity of the employed assay asprosin levels in body fluids may not be detectable without pre-concentration. To selectively concentrate asprosin from body fluids we generated an affinity column by immobilizing pc-asp via CNBr to sepharose beads (Fig. 3A). After sample application, the column was washed and the captured amounts of asprosin were eluted at pH 2.5 and neutralized (see “Materials and Methods” section). Using this approach, we were able to selectively pull-down asprosin from the cell culture supernatant of RPE cells (Fig. 3B). ELISA and SPR analysis revealed an eight-fold increase of asprosin concentration by using this affinity column approach (Fig. 3B). With this approach, we were able to obtain sufficient serum asprosin amounts for western blot detection. Thereby, we could demonstrate that human asprosin circulating in serum has a molecular weight of around 40 kDa (Fig. 3C), similar to the recombinantly expressed asprosin (Fig. 1B). Utilizing the pc-asp affinity column approach allowed us also to concentrate asprosin from urine, for the first time. However, after concentration of 200 ml urine, we were able to detect about 3 ng/ml of asprosin which corresponds to 15 pg/ml present in non-concentrated urine (Fig. 3D).

### Measurement of asprosin in human cell lines and clinical samples

#### Secreted amounts of asprosin by human cell lines depend on tissue origin

**Figure 4:**
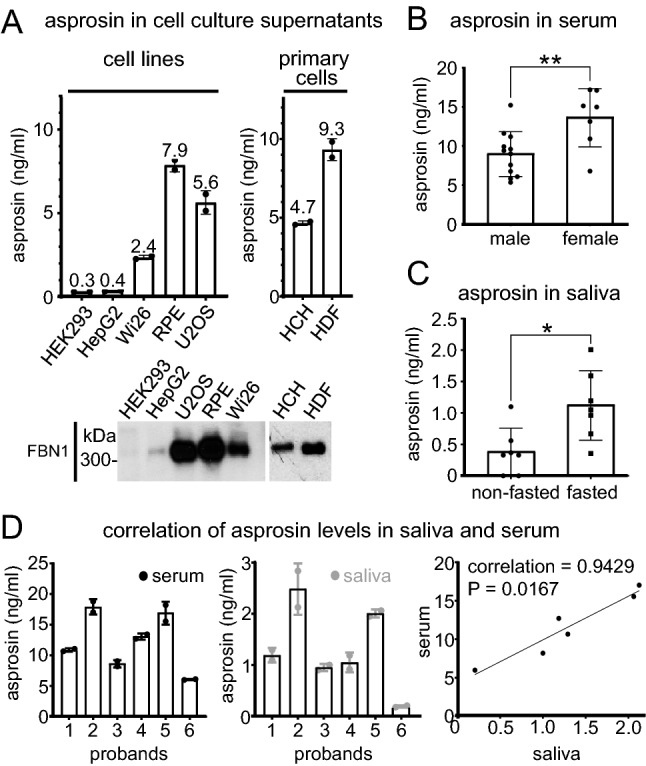
Asprosin levels in serum and saliva depend on biological sex, fasting, and show a strong correlation. (**A**) (top left) Endogenous asprosin levels in various human cell lines supernatants and (top right) primary human cells (human dermal fibroblasts and human chondrocytes) were measured using asprosin sandwich ELISA (n = 2 per each group). (bottom) western blot showing the corresponding fibrillin-1 expression in cell lines supernatants. (**B**) Comparative analysis of asprosin levels in blood serum (25 µl serum fourfold diluted in 75 µl PBS) of male and female groups (n = 19, m/f = 12/7, age = 30.4 ± 6.4 years, weight (W) = 72.9 ± 11.3 kg, height (H) = 1.75 ± 0.08 m, BMI = 23.7 ± 2.9 kg/m^2^) using sandwich ELISA and analyzed by unpaired two-tailed t-test, **P = 0.0072. (**C**) Assessment of asprosin levels in saliva (100 µl saliva) in fasting (overnight fasting, 8–9 h) and non-fasting (3 h after lunch) conditions by sandwich ELISA (n = 7, m/f = 3/4, age = 32.3 ± 8.71 years, weight = 70.5 ± 13.8 kg, height = 1.74 ± 0.09 m, BMI = 23.3 ± 3.04 kg/m^2^) and analyzed by Wilcoxon matched-pairs signed-rank test, *P = 0.0156). (**D**) (left and middle) Quantification of asprosin levels in human serum and saliva respectively in fasted condition (overnight fasting) by sandwich ELISA (n = 6, m/f = 3/3, age = 32.7 ± 9.09 years, weight = 64.5 ± 8.77 kg, height = 1.71 ± 0.07 m, BMI = 22.1 ± 2.44 kg/m^2^). (right) Correlation of asprosin levels in human serum and saliva using Spearman correlation = 0.94, *P = 0.02, (n = 2 per each group). Data were analyzed using Graphpad Prism version 8.0.2.

To test our established sandwich ELISA in complex solutions, we measured asprosin protein amounts secreted by several human cell lines into the conditioned media. Our results showed that cell lines derived from metabolically active organs such as kidney (HEK293: embryonic kidney) or liver (HepG2: liver cancer) secreted about 20-fold less asprosin into the cell culture supernatant when compared to cells derived from connective tissues such as bone (U-2 OS: bone osteosarcoma), eye (RPE: retinal pigment epithelial), lung (WI-26: fetal lung), cartilage (HCH: primary human chondrocytes derived from knee cartilage) or skin (HDF: primary human dermal fibroblasts) (Fig. 4A). However, the detected asprosin amounts correlated with the secreted amounts of fibrillin-1 which were significantly higher in cells derived from extracellular matrix (ECM) protein-producing connective tissues (Fig. 4A, bottom).

#### Asprosin levels in serum and saliva correlate strongly and depend on biological sex and feeding status

To test the accuracy of our sandwich ELISA in clinical samples we performed spike-and-recovery and linearity-of-dilution experiments in serum and plasma (Supplementary Fig. S6). The signal recovery was not affected when our recombinant asprosin standard was serially diluted in human plasma or serum (Supplementary Fig. S6). Measurement of serum samples from age, biological sex, and BMI matched individuals showed that asprosin levels were significantly higher in women than men (Fig. 4B). It was previously reported that asprosin serum levels increase upon fasting^1^ and that asprosin can also be detected in saliva^16^. We were therefore interested whether a fasting-induced increase of asprosin can be also detected in saliva, and whether serum and saliva levels show a correlation. Assessment of saliva samples from the same individuals 3 h after lunch (non-fasted) or after overnight fasting (fasted) showed that fasting induced an increase of asprosin levels in saliva (Fig. 4C). We then quantified asprosin amounts in simultaneously taken serum and saliva samples from several individuals after overnight fasting and performed a Spearman correlation analysis. Our results revealed a strong correlation (Spearman correlation coefficient: ρ = 0.9429, *P = 0.0167) between asprosin levels in serum and saliva (Fig. 4D). Asprosin levels in serum and plasma from the same individuals were about ten-fold higher than in saliva. Similar to the low asprosin concentrations detected in saliva, we detected asprosin in human breast milk in the range of (1.1–3.3 ng/ml). An overview of the ranges of detected asprosin in human body fluids is given in Table 1.

**Table 1:** Ranges of determined asprosin concentrations (mean ± SD) in various body fluids.

Sample	f/m	Age (years)	BMI (kg/m^2^)	Asprosin (ng/ml)
Serum	7/5	31.6 ± 7.3 (27–36)	23.2 ± 3.8 (21–26)	14.7 ± 8.9 (9.1–20.4)
Plasma	7/5	31.6 ± 7.3 (27–36)	23.2 ± 3.8 (21–26)	11.5 ± 3.9 (8.9–13.9)
Saliva	8/6	30.2 ± 6.6 (26–34)	23.4 ± 3.7 (21–26)	1.07 ± 0.9 (0.5–1.6)
Breast milk	9/0	33.1 ± 4.9 (29–37)	23.4 ± 4.1 (20–27)	2.2 ± 1.4 (1.1–3.3)

### Measurement of asprosin in patient cohorts with documented cartilage turnover

#### Asprosin serum levels increase after 30 min of running exercise

**Figure 5:**
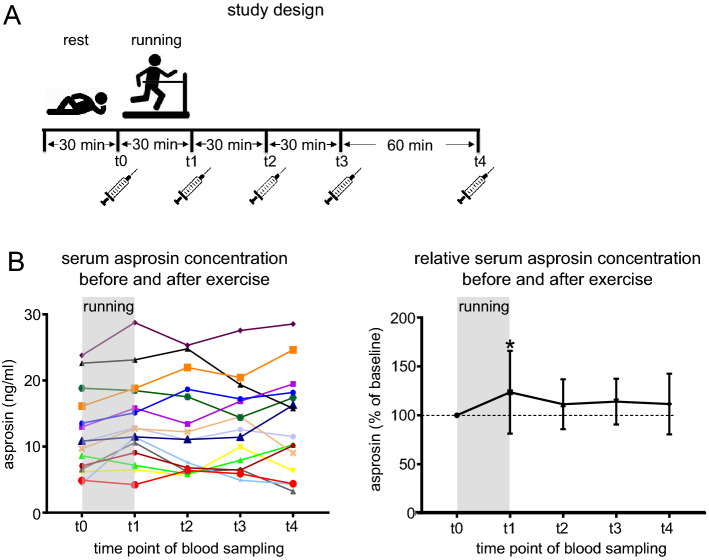
Asprosin serum levels increase after 30 min of running exercise. (**A**) Schematic diagram representing the overall study design showing exercise and rest regimens and blood sampling time points. (**B**) (Left) Absolute serum asprosin concentration (ng/ml) for each subject before and after running exercise (n = 15 subjects); t0: immediately before running exercise (after 30 min rest in sitting position); t1: immediately after exercise; t2, t3, and t4 represent 30 min, 60 min, and 120 min after exercise, respectively. (right) Relative serum asprosin concentration after running exercise (t1, t2, t3, and t4) normalized to its baseline concentration before exercise (t0) and represented as percentage. A significant increase of asprosin concentration was observed immediately after running exercise; asprosin concentration (c) at t0 = 100%; c(asprosin) t1/c(asprosin) t0 = 123.7 ± 42.3%; c(asprosin) t2/c(asprosin) t0 = 111.3 ± 25.5%; c(asprosin) t3/c(asprosin) t0 = 114 ± 23.40%; c(asprosin) t4/c(asprosin) t0 = 111.5 ± 31.06%; *P = 0.0385. Serum (25 µl) fourfold diluted in PBS (75 µl) for asprosin concentration analysis. Data were analyzed using Graphpad Prism version 8.0.2.

Recent studies reported that asprosin serum levels are regulated by physical activity^17,18^. For instance, it was found that plasma asprosin levels were elevated only in women upon 20 s of anaerobic exercise^19^. To further investigate whether physical exercise affects asprosin blood levels in men, and whether cartilage may be a potential source of serum asprosin we measured serum levels of asprosin before and after running exercise in a cohort for which release of the cartilage degradation marker cartilage oligomeric matrix protein (COMP) was already reported^20^. 15 young male athletes (age 27.5 ± 3.1 years, Supplementary Table S7) were subjected to 30 min running exercise. Blood sampling was performed immediately before (after 30 min rest in supine position) and after the 30 min running exercise. Further blood samples were taken 30, 60, and 120 min after the end of the running exercise (Fig. 5A). Serum asprosin levels increased by about 25% on average (123.7 ± 42.3%) immediately (t1) after 30 min running exercise and remained at an elevated but not significant level (12.5% increase) for at least 120 min after (111.5 ± 31.06%) (Fig. 5B). An overview of the determined asprosin serum levels determined in this cohort is given in Supplementary Table S4.

#### Asprosin levels in serum correlate with the cartilage marker COMP upon hip replacement surgery

**Figure 6:**
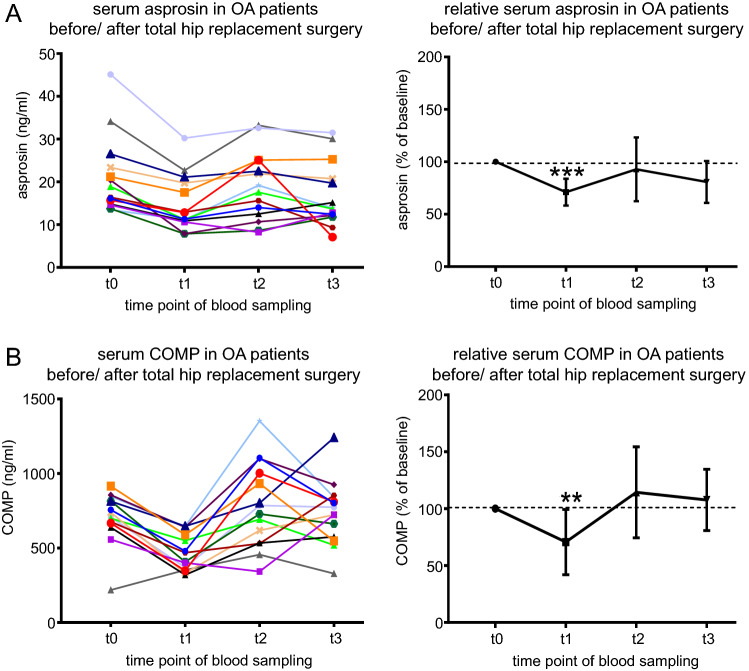
Asprosin levels in serum correlate with the cartilage marker COMP upon hip replacement surgery. (**A**) (left) Absolute serum asprosin (ng/ml) for each patient before and until one year after total hip replacement (THR). (right) Relative asprosin concentration after THR (t1, t2, and t3) normalized to serum asprosin at baseline (t0, before THR). (**B**) (left) Absolute serum COMP (ng/ml) for each patient before and until one year after THR. (right) Relative serum COMP (%) after THR (t1, t2, and t3) normalized to serum COMP at baseline (t0, before THR). n = 14 patients; time points: t0: within 1 week before THR, t1: 7 days postoperative, t2: 3 months postoperative, t3: about 1 year postoperative. ***P = 0.0003, **P = 0.0056. Serum (25 µl) fourfold diluted in PBS (75 µl) for asprosin concentration analysis, and 2 µl serum 50-fold diluted in 98 µl PBS for COMP concentration analysis. Data were analyzed using Graphpad Prism version 8.0.2.

Since we found asprosin to be secreted by primary human chondrocytes (Fig. 4A), we were interested whether serum asprosin levels correlate with cartilage related pathologies. Therefore, we measured serum asprosin levels of patients (n = 14) diagnosed with symptomatic hip osteoarthritis (OA) prior and after total hip replacement (THR) surgery (Fig. 6). We previously reported that COMP, a biomarker of cartilage degeneration and osteoarthritis showed a reduction, one week (t1) after hip joint replacement surgery, and returned to pre-operative serum levels three months (t2), and one year (t3) post-surgery^21^. Our measurements revealed that serum asprosin levels strongly correlated with COMP levels, showing a 50% reduction at one week post- surgery before reaching 90% of pre-operative levels after three months (t2) and up to one year (t3) post-surgery (Fig. 6A,B, Supplementary Table S5, and S6).

### Analysis of asprosin localization suggests its storage in chondrocytes and cartilage tissue

#### Asprosin localization in primary human chondrocyte culture

**Figure 7:**
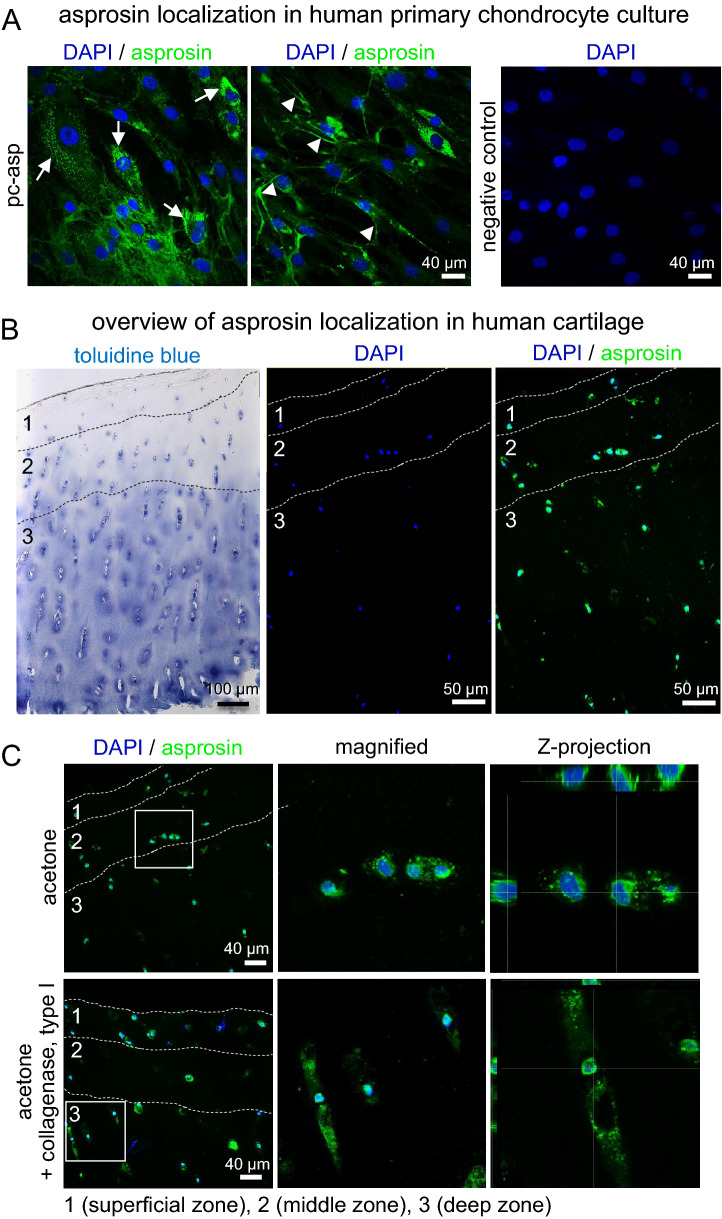
Asprosin localization in primary chondrocyte cultures and cartilage tissue. (**A**) (left and middle) Confocal immunofluorescence microscopy of asprosin in primary human chondrocytes. Cells were incubated with pc-asp antibody (green) and DAPI (blue). Endogenous asprosin signals were predominantly observed as two staining patterns: (left, white arrows) vesicle-like structures, and (middle, white arrow heads) cytoplasmatic signals with diffused distribution that significantly accumulated in cell projections forming fiber-like structures. (right) Secondary antibody only staining (negative control). These two staining patterns were simultaneously detected in the majority of chondrocytes after 11 days in culture. Shown images are representative of two independent experiments. (**B**) (left) Representative toluidine blue stained human articular cartilage tissue illustrating approximate thickness of the different zonal regions. (middle and right) Detection of asprosin in chondrocytes within human cartilage by confocal immunofluorescence microscopy. Overview image of cryosection from cartilage specimen fixed with acetone and incubated with pc-asp (green) and DAPI (blue) showing asprosin expression in different cartilage zones. (C) Comparative analysis of asprosin signals after acetone fixation (top panel), or acetone fixation and additional collagenase type I treatment (bottom panel). Sections were incubated with pc-asp antibody (green) and DAPI (blue). The antibody revealed both diffuse cytoplasmatic signals and vesicle-like structures of asprosin as shown in overview and magnified areas (3.5-fold, marked by white boxes) under both conditions. (right) Z-projections show a prominent perinuclear and pericellular distribution of the asprosin signal. Remarkably, additional collagenase type I treatment resulted in a wider signal distribution in chondrocyte associated areas as well as in all cartilage zones. Toluidine blue staining was imaged with Zeiss AX10 light microscope and Diskus software (version number 5.0.6353 #7599). Confocal images were obtained from a Leica SP8 confocal microscope and Leica LAS AF Lite 4.0 software. Images were further processed using Fiji/ImageJ software to obtain average intensity Z-projection.

To further investigate the possibility of local asprosin storage in cartilage, primary human chondrocyte cultures were analyzed by immunofluorescence using pc-asp antibody (Fig. 7A). In primary human chondrocytes asprosin showed predominantly two staining patterns: a slight diffuse signal distributed in cytoplasm and in cytoplasmic processes, and intensive vesicle like structures, distributed predominately in the cytoplasm (Fig. 7A). In most cells, both types of signals were observed simultaneously. However, an increase in signal intensity and the formation of fiber-like structures were observed in the periphery of some cytoplasmic processes (Fig. 7A), whereas less closely spaced vesicle-like structures were also detected around or in the vicinity of the cells.

#### Asprosin localization in human cartilage

Furthermore, we investigated asprosin localization in human cartilage. The immunoreactivity of pc-asp antibody was observed, independent of the used fixatives, as an intensive signal localized to chondrocytes and their pericellular microenvironment^22^ (Fig. 7B, Supplementary Fig. S7). The immunoreactivity was detected in chondrocytes distributed throughout all cartilage zones. Close inspection of chondrocytes in acetone treated cryosections revealed, similar to the analysis of cultured primary chondrocytes, diffuse distributed signals surrounding the nuclei and vesicle like structures. Accumulation of vesicle like structures was observed perinuclear or in the area comparable to the lacunar space. It is noteworthy that scattered vesicles were detectable, albeit to a much lower degree and signal intensity, in the interterritorial regions. Compared to only acetone treated cryosections, the signal intensity in acetone-metanol fixed specimens appeared weaker. In 4% PFA fixed cryosections a predominantly diffuse staining pattern was seen (Supplementary Fig. S7). To increase the availability of pc-asp epitopes, we performed enzymatic antigen retrieval with empirically selected proteases. For this purpose, digestion solutions containing pepsin, hyaluronidase, proteinase K and their combination or collagenase type 1 were applied to acetone-treated sections. As expected, treatment of cartilage sections with the different enzymes separately or in combination resulted in a broader signal distribution of asprosin within chondrocyte associated areas. The strongest intensity and the largest area of chondrocyte associated asprosin signal distribution were observed when sections were treated with pepsin or enzyme mixtures (Supplementary Fig. S8). However, enzyme treatment resulted in a low preservation of morphological structures. When the sections were treated with collagenase type 1, a better preservation of cell nuclei and a significant increase of the asprosin signal were observed. Analysis of collagenase type 1 treated sections at higher magnification and in Z-stacks showed clear perinuclear signal accumulation while accumulations or clusters of vesicles-like structures distributed in the periphery of nuclei revealed a staining pattern comparable to chondrons^22^ (Fig. 7C).

## Discussion

Since its discovery as a new metabolic hormone, several clinical studies have been conducted to establish a correlation of asprosin levels with obesity, diabetes mellitus, insulin resistance, metabolic syndrome, or diabetic nephropathy^4,6^ (Supplementary Table S1). Pathologically elevated asprosin levels were reported in patients with obesity^7,16^, insulin resistance^23,24^, and diabetes mellitus type 1^25^ and type 2^26–28^. Therefore, current research attempts focus on the possibility of using anti-asprosin antibody therapy to regain metabolic health^3^. However, the considerable variability of reported asprosin levels in human serum, plasma, and saliva, ranging from < 0.5 to > 350 ng/ml raises serious concerns regarding the reliability of sensitive asprosin measurements (Supplementary Table S1). Without the availability of reliable methods for asprosin detection in clinical samples any informative correlation studies or future administration of effective anti-asprosin therapy remain futile. This prompted us to establish new biochemical approaches for the sensitive and reliable measurement of asprosin in clinical samples.

Analysis of human asprosin produced by human embryonic kidney cells revealed a significant amount of N-terminal glycosylation similar to physiological asprosin circulating in human blood (Fig. 3C) which contributes to about 50% of its molecular mass. As shown in yeast^14^, our analysis demonstrated that all three predicted N-glycosylation sites (N_3_, N_19_, and N_36_) were utilized (Supplementary Fig. S3). Our analysis also showed that additional O-glycosylation of asprosin is likely (Supplementary Fig. S2). Currently, the function of asprosin glycosylation is not clear, however, similar to other proteins it is possible that it keeps asprosin more soluble and may therefore prevent its aggregation in blood^29^. By employing our newly raised polyclonal asprosin antibody we established sensitive direct and sandwich ELISAs with a reliable detection below 0.15 ng/ml which is comparable to the stated sensitivity of commercially available ELISA kits used in previous studies^5^ (Supplementary Table S1). The high affinity of the generated pc-asp (K_D_ ~ 300 pM) also enabled us to selectively concentrate asprosin from complex clinical samples via antibody affinity chromatography. Using this approach we were able to determine asprosin in urine for the first time (Fig. 3D) and retrieved sufficient amounts from serum for subsequent detection by western blotting (Fig. 3C).

Our measurements suggest the following range of asprosin levels in clinical samples: serum (9–20 ng/ml), plasma (9–14 ng/ml), saliva (0.5–2 ng/ml), and breast milk (1–3 ng/ml) (Table 1). Asprosin serum and plasma levels determined by us are in agreement with ranges previously measured in large patient cohorts with over 100 individuals^30–32^ (Supplementary Table S1), suggesting that these ranges represent physiological relevant asprosin concentrations. Our investigations also provided a possible explanation for the strong deviation of reported serum and plasma asprosin levels by other studies (Supplementary Table S1). The first report about blood asprosin levels was by Romere et al.^1^ who employed similar to our sandwich ELISA, a combination of polyclonal and mouse mab (capture: mouse mab against human profibrillin amino acids 2838–2865; detector: polyclonal goat against human profibrillin amino acids 2737–2750 by Abnova). However, two additional studies using the same detector but different capture antibodies reported serum asprosin levels ranging from 10–220 to 307–7454 ng/ml^7,33^. This indicates that the choice of the capture antibody is critical for obtaining reliable asprosin measurements. Also using non-glycosylated recombinant asprosin produced in *E. Coli* for standard curve^1^ generation may result in artificially skewed concentration ranges. Our biochemical investigations showed that glycosylation of asprosin may affect detection sensitivity in ELISA and western blot analysis (Fig. 1G, Supplementary Fig. S5H,I). For instance, ELISA detection sensitivity of deglycosylated asprosin by mab (clone Birdy-1) appeared to be increased (Supplementary Fig. S5H, right). Therefore, it is plausible that differences in detection sensitivity between *E.Coli* produced asprosin standards and glycosylated physiological asprosin are the underlying cause for the enormous variance of reported asprosin values. Despite 92% sequence identity between murine and human asprosin, our polyclonal antibody raised against folded and glycosylated full length human asprosin did not cross-react with murine asprosin (Supplementary Fig. S5D). Also, we so far failed to reliably detect asprosin amounts in mouse serum or plasma samples by using commercially available sandwich ELISA kits (unpublished results). This suggests that the availability of surface epitopes for human and murine asprosin differs. Overall, our data indicate that reported absolute asprosin levels in humans and mice should be considered with caution. For instance, it was reported that plasma asprosin levels display a circadian oscillation in mice and vary from 8 to 14 nM^1^. However, similar human and murine plasma asprosin levels in the range of 5–14 nM as reported by Romere et al.^1^ would correspond to 185–440 ng/ml which are at least one order of magnitude higher compared to our determined values (Table 1) and those recently measured in larger cohorts (Supplementary Table S1).

Our developed sandwich ELISA showed sufficient sensitivity to reliably detect asprosin at lower amounts in the range of 1–2 ng/ml. This allowed us to measure asprosin in breast milk, for the first time. Asprosin levels in breast milk might be an indicator of the metabolic health status of the mother, however, it may also contribute to the mother-infant communication. In the context of perinatal programming, assessing asprosin levels in breast milk might be informative, since it could be shown that other metabolic hormones like leptin, ghrelin, insulin and GLP-1 are present in breast milk and frequently correlate with the maternal BMI^34^. Therefore, asprosin concentrations in breast milk may also depend on the maternal BMI and elicit metabolic effects in the neonate.

Our serum measurements showed that asprosin was significantly elevated in females (Fig. 4B), which is similar to previous findings^35^. Currently, there is only limited information available regarding sex-dependent expression rates of fibrillin-1 from which asprosin is derived after furin-mediated cleavage within the C-terminal region. However, analysis of fibrillin-1 mutant mice revealed sex-dependent differences in thoracic aorta contractility, as well as aortic media injury^36,37^. Interestingly, addition of 17β-estradiol was found to promote fibrillin-1 production by human aortic smooth muscle cells, but not by fibroblasts^38^, suggesting a sex- and tissue-dependent regulation of fibrillin-1 expression which may explain the observed increase of asprosin in serum samples from females.

Saliva asprosin concentration is also increased upon fasting which strongly correlated with serum asprosin levels (Fig. 4C,D). Due to the high sensitivity and low intra- and inter-assay variability compared to commercially available asprosin detection kits (Supplementary Table S1), our established sandwich ELISA may be routinely employed for the analysis of saliva as useful alternative to serum samples as its collection is simple, non-invasive and painless^39,40^. Recently, a correlation of saliva with serum asprosin was already demonstrated and seems to be BMI-dependent^16^. However, the reported asprosin levels in saliva from donors with normal BMI similar to our tested cohort was reported to be around 30 ng/ml, which is 30-times higher than what we observed on average under fasting conditions (Fig. 4C, Table 1). Also recently, asprosin saliva amounts were reported to be about 25 ng/ml in a normal control group using the same commercially available ELISA kit^41^. Unfortunately, the kit used by both studies (Sunred Bioscience) is no longer commercially available, so that detailed information on its components and characteristics could not be retrieved.

There has been conflicting evidence reported regarding the effect of exercise on asprosin blood levels. The first report stated a sex-dependent increase of asprosin upon acute anaerobic exercise^19^. Serum levels of asprosin were found to be elevated only in women 15 min, 30 min, 60 min, and 24 h after a 20 s bicycle exercise. However, aerobic exercise in diabetic male rats led to a decrease of hepatic asprosin levels^42^. Recently, a significant decrease in serum asprosin was also observed in adult male individuals subjected to an aerobic exercise protocol in moderate intensity for 30 min at two different time periods of the day (morning: 08:00–10:00 h, evening: 20.00–22.00 h) at least 3 d apart. A significant decrease in asprosin serum levels was observed in normal and overweight, or obese participants of the study^18^. However, a comparison of absolute blood asprosin amounts determined in both studies raises concern about the informative value of the data. While Wiecek et al.^19^ report plasma asprosin levels in the range of 106–362 ng/ml, Ceylan et al.^18^ report serum asprosin levels within 0.53–1.17 ng/ml (Supplementary Table S1). Recent studies conducted with large cohorts including > 100 BMI, age, and biological sex matched controls showed that values for serum and plasma asprosin are in the same range as determined by our measurements (13–32 ng/ml) (Table 1 and Supplementary Table S1), but out of the reported ranges by both previously published exercise studies^18,19^.

Our findings show a significant increase of asprosin after 30 min of running exercise (Fig. 5). Before the experiment, which took place in the morning, subjects were asked not to exercise for 24 h. Participants remained seated for 30 min before the first blood sample (baseline) was drawn. Our results clearly showed that upon acute running exercise asprosin release into the blood is induced and remains stable for at least 2 h afterwards. So far it is not understood by which mechanism running exercise leads to increased asprosin levels. Previously, plasma asprosin levels were reported to be increased upon a 20 s bicycle sprint in women^19^ which represents an exercise regime with less mechanical loading compared to treadmill running. Since these increased asprosin levels upon a low impact bicycle sprint also correlated with irisin, an exercise-induced myocyte produced cytokine^43^, and fibrillin-1 is known to be ubiquitously expressed in connective tissues including skeletal muscle^1,44^, muscle tissue was suggested as source of asprosin secretion^19^. However, since irisin and asprosin are also produced by adipocytes^1,45^ it remains difficult to assess the tissue-specific contribution to circulating asprosin levels in blood upon exercise. Measurement of asprosin in combination with tissue-specific markers may allow dissecting which musculoskeletal compartment might be challenged by a certain exercise regimen and activate asprosin synthesis or release. In this context, it is also not clear how the applied mechanical impact due to the chosen exercise regimen may be implicated in altered asprosin synthesis or release. Mechanical loading could result in cellular adaptation leading to anabolic reactions and asprosin synthesis. Alternatively, mechanical loading may lead to connective tissue degradation and thereby cause release of tissue resident asprosin into the circulation. Mechanical loading of joints due to exercise leads to degradation of cartilage and release of cartilage extracellular matrix components such as COMP into the serum^20,46^. We previously reported that COMP levels increased upon running exercise in the same cohort^20^. If asprosin is a component of cartilage and produced by chondrocytes (Fig. 4A), exercise induced break down of cartilage may also correlate with cartilage degradation markers such as COMP and contribute to serum levels. To test this hypothesis we analyzed asprosin and COMP levels in OA patients before and after THR (Fig. 6). Our analysis showed a clear correlation of asprosin and COMP serum levels in both the exercise cohort and the THR cohort suggesting asprosin as component of cartilage, for the first time. Fibrillin-1 is known to be a component of cartilage and where it assembles into supramolecular microfibrils that structurally contribute to the cellular microenevironment of chondrocytes^47^. It is therefore possible that also aprosin is locally stored within cartilage and released upon mechanical loading.

To further investigate the idea of local asprosin storage in cartilage, we analyzed asprosin localization in primary human chondrocyte culture and cartilage tissue (Fig. 7). Close inspection of the signal distribution in cultured primary chondrocytes and in chondrocytes embedded in cartilage tissue revealed two similar staining patterns that appeared as diffuse signals in the cytoplasm and in cytoplasmic processes, as well as vesicle-like structures (Fig. 7). Interestingly, in the periphery of some cytoplasmic processes detected signals were accumulated to fiber-like structures (Fig. 7A). Cytoplasmic processes in chondrocytes were described to be formed as active chondrocyte responses due to changes in the ECM that can result in release of cytokines or degradative enzymes^48^. It appears therefore plausible that asprosin may be released into the extracellular space upon mechanical loading. Within each zone of articular cartilage the composition of the ECM is region-specific^49^. This property of the matrix can significantly influence the results of immunohistochemistry, for example by inhibiting the penetration of antibodies^50^. We therefore performed additional pretreatment of cartilage sections with different enzymes partially removing collagen fibers or proteoglycans. Enzyme treatment indicated that collagens, hyaluronan and other ECM components contribute to masking of asprosin detection (Fig. 7C). However, this finding still supports the hypothesis that asprosin is secreted by chondrocytes and deposited within their pericellular matrix.

Our results support the idea of local asprosin storage within tissue-specific microenvironments. Potential extracellular storage platforms could be proteoglycans or other ECM supramolecular scaffolds such as the fibronectin/elastic fiber network which have been known to act as sinks or targeting scaffolds for connective tissue derived growth factors^11,51^. Asprosin stored in tissues may function as sensor for local energy demand. Experiments with murine myoblasts (C2C12) showed that asprosin is not only able to interfere with muscle cell insulin sensitivity^52^, but also up-regulates glucose transporter 4 (GLUT4) expression in C2C12 myotubes and thereby enhance local glucose uptake by tissue muscle cells^14^.

More extensive studies are required to investigate how asprosin is released into different body fluids and what exact function it serves there. Also, the mechanisms of asprosin release upon exercise require further investigation, especially considering different exercise regimen from low to high mechanical loading. Another important question is whether asprosin release is influenced by sex hormone levels which may explain the sex-specific differences we observed. Overall, our results suggest that reported absolute asprosin levels in clinical samples have to be considered with caution, since used commercially available ELISAs show a considerable variation in detection sensitivity. Our biochemical approaches and generated tools will now allow in-depth analysis of asprosin biological functions on a molecular level.

## Materials and methods

### Ethics statement

All research on human tissues was performed in accordance with local regulations and was approved by the Human Ethics Committee of the University Hospital of Cologne (No. 18/124, No. 15/368, No. 14-422), the local ethics committee of the Goethe University Frankfurt in Germany (No. 497/15) and the German Sport University Cologne Germany^20^. Written Informed consent was obtained from all participating probands and patients who agreed to have materials examined for research purposes. The study was conducted in accordance with the Declaration of Helsinki.

### Expression and purification of proteins

Human asprosin (S^2732^-H^2871^) and human placensin (S^2780^-Y^2912^) DNA sequences were generated by GeneArt Strings DNA Fragments service (Thermo Fisher Scientific, Massachusetts, USA) including the restriction enzymes sequences for NheI at the 5′ and XhoI at the 3′ ends. Double strained synthetic cDNAs encoding for the respective protein sequences were cloned via the Nhe1/Xho1 sites of the modified pCEP-Pu vector containing an N-terminal BM-40 signal peptide and a C-terminal double Strep-tag II downstream of the Xho1 restriction site^53^. Ligated vectors were transformed into One Shot TOP10 Chemically Competent E. coli (Thermo Fisher Scientific, Massachusetts, USA), and clones were verified by Sanger sequencing (Microsynth Seqlab GmbH, Göttingen, Germany). HEK-293 EBNA cells cultured in Dulbecco’s modified eagle medium (DMEM) (Thermo Fisher Scientific, Massachusetts, USA) were transfected with asprosin or placensin expression vectors by using FuGENE HD Transfection Reagent (Promega GmbH, Germany) according to the manufacturer’s instructions. One day after transfection, cells were selected by supplementing the medium with puromycin (1 µg/ml). The cells were expanded for large scale production in TripleFlask Cell Culture Flasks (Thermo Fisher Scientific, Massachusetts, USA). At 90% confluency, cells were incubated with serum-free DMEM containing puromycin (0.5 µg/ml) and collected after 48 h. Collected medium was filtered with a suitable membrane filter, then subjected to Strep-Tactin XT gravity flow columns (2 ml beads; IBA GmbH, Germany) at 4 °C overnight. Asprosin or placensin were eluted with elution buffer (100 mM Tris/HCl, pH 8.0, 150 mM NaCl, 1 mM EDTA, 2.5 mM desthiobiotin). The collected fractions were analyzed by Coomassie Blue Staining, concentrated and the buffer exchanged to PBS by using Amicon Ultra Centrifugal Filters (cut-off: 3 kDa) (Merck Millipore, Burlington, MA, USA). The N-terminal half of fibrillin-1 (rF90, amino acids positions: M^1^-V^1527^) with a C-terminal His_6_-tag was overexpressed in HEK-293 EBNA cells followed by purification as described previously^54^. C-terminal half of fibrillin-1 (rF6, amino acids position: amino acid positions V^1487^-H^2871^) was overexpressed in HEK-293 EBNA cells. The overexpression construct for rF6^55^ was a kind gift from Lynn Sakai, Oregon Health and Science University, Portland, OR, USA. Recombinant human (#761902) and mouse (#762002) asprosin were purchased from Biolegend (San Diego, CA, USA).

### Antibodies

The pc-asp anti-asprosin antibody was generated against full-length human asprosin. Recombinantly expressed human asprosin was used to immunize a rabbit for antibody production (Pineda Antibody Service, Berlin, Germany). Preimmune serum (0.5 ml) was obtained before the immunization of the rabbit and did not show cross reactivity to asprosin tested by ELISA. The rabbit was immunized with a total amount of 500 µg asprosin in five consecutive immunizations at days 1, 14, 28, 42, and 56. The antiserum was obtained 60 days after immunization. The antiserum was affinity purified on a column of CNBr-activated Sepharose 4B (Cytiva, Uppsala, Sweden) conjugated with the recombinantly expressed asprosin (300 µg) according to the manufacturer's instructions. The antibody was eluted with 0.1 M glycine (pH 2.5) and neutralized with 3 M Tris/HCl, pH 8. The eluted antibody was concentrated by using Amicon Ultra Centrifugal Filters (cut-off: 10 kDa), (starting volume: 6 ml, end volume: 1 ml). The antibody was used as a capture antibody for sandwich ELISA at 3 µg/ml, at a 1:5000 dilution (0.2 µg/ml) for western blot, and at a 1:70 dilution (14 µg/ml) for cells and tissues immunofluorescence. Mab anti-asprosin antibody (clone Birdy-1¸ AG-20B-0073) (AdipoGen Life Sciences Inc., San Diego, USA) was used at a 1:1000 dilution (1 µg/ml) for western blot and at a 1:2000 dilution (0.5 µg/ml) as detection antibody in sandwich ELISA. Fibrillin-1 polyclonal antibody, raised against C-terminally His_6_-tagged rF90 representing the N-terminal half of fibrillin1^51^, was used at a 1:5000 dilution (0.5 µg/ml) for western blot. Fibrillin-1 rabbit monoclonal antibody (CPTC-FBN1-3) (DSHB, Iowa, USA), raised against the synthetic peptide: TCVDINECLLEPR, which is contained within the C-terminal half of fibrillin-1, was used at a 1:2500 dilution (1 µg/ml).

### Protein quantification

Concentrations of proteins, which were used in this study including pc-asp antibody were colorimetrically measured by BCA Protein Assay Kit (Thermo Fisher Scientific, Massachusetts, USA). In addition to the BCA assay, the concentrations of purified asprosin and placensin were spectrophotometrically measured by NanoDrop One (Thermo Fisher Scientific, Massachusetts, USA) after buffer exchange into PBS using Amicon Ultra Centrifugal Filters (cut off: 3 kDa). The protein quantification was performed using the program “Protein A280” which measures the protein absorbance at 280 nm and calculates the concentration depending on the protein extinction coefficient. The predicted extinction coefficient for human asprosin-2 × Strep-tag II: 25,440 M^−1^ cm^−1^, and human placensin-2 × Strep-tag II: 19,940 M^−1^ cm^−1^, were obtained by using the ProtParam tool provided on the ExPASy Server.

### Cells

HEK293 EBNA and HEK293 (adherent epithelial cells of human embryonic kidney/ATCC CRL-1573), U2OS (adherent osteosarcoma cells from human bone/ATCC HTB-96), WI-26 (human lung fibroblast cell line/ATCC CCL95), hTERT RPE (adherent human retinal pigment epithelial cell line/ATCC CRL-4000), Hep G2 (adherent human liver cancer cell line/ATCC HB-8065), and HDF (primary human dermal fibroblasts) as well as HCH (primary human chondrocytes) were established from biopsies. Cells were cultured in DMEM with 10% fetal calf serum (FCS) and 1% penicillin/streptomycin, they were grown in incubators maintained at 37 °C and 5% CO_2_. The cells were fed twice per week and split regularly.

### Affinity chromatography with pc-asp column

Affinity purified pc-asp anti-asprosin antibody was coupled to CNBr-activated Sepharose 4B beads according to the manufacturer’s instructions. 500 µg of purified pc-asp anti-asprosin antibody dialyzed in 0.1 M NaHCO_3_, pH 8.3 containing 0.5 M NaCl (coupling buffer) was incubated with 0.5 ml CNBr agarose beads and the mixture was kept rotating at 4 °C overnight. The uncoupled sites on beads were blocked with 0.1 M Tris–HCl, pH 8.0 for 2 h at RT. Poly-Prep Chromatography Columns (Bio-Rad; Hercules, USA) were packed with uncoupled CNBr beads (negative control column) and pc-asp anti-asprosin antibody coupled CNBr beads (asprosin pull-down column). The two columns were connected in series respectively, and then samples were applied to the two columns pull-down system. Prior to application, 1 L serum-free cell culture supernatant from RPE cells or 200 ml urine were filtered through a 0.22 μm filter unit (Sterile Steritop Bottle, Merck Millipore, Darmstadt, Germany) followed by pH adjustment to 7.5 with 5 M NaOH solution. For the application of serum samples, 1 ml of serum was diluted 1:10 with 10 mM Tris–HCl, pH 7.5, and filtered through a sterile 0.22 µm syringe filter. After the samples were loaded and passed through the columns at 4 °C, the columns were washed with 100 ml of 10 mM Tris–HCl, pH 7.5. Recovery of bound proteins in the negative control and asprosin pull-down columns was done by elution in six column volumes 0.1 M Glycine (pH 2.5) and neutralization of the elution fractions with 3 M Tris–HCL, pH 8. Eluted fractions were concentrated followed by buffer exchange using Amicon Ultra Centrifugal Filters with a molecular cut-off of 3 kDa. Concentrated elution fractions were subjected to SDS-PAGE followed by Coomassie staining, western blotting, or SPR.

### Enzymatic cleavage of N-glycans

The sample was placed into an Amicon Ultra centrifugation device with a 10 kDa cut-off and centrifuged at 14,000 × g (4 °C) for 10 min to exchange the buffer (0.1 M ammonium bicarbonate buffer, pH 8.0). To 20 µg asprosin in 20 µl buffer a 0.5 µl aliquot of PNGaseF (BioLabs, 250 units, containing glycerol) was added and incubated for 16 h at 37 °C. The sample was dried by vacuum rotation and taken up in 0.1% aqueous trifluoroacetic acid (100 µl) before solid-phase extraction on C18 (see below).

### Solid-phase extraction of de-N-glycosylated peptides

The cartridge was activated with 80% acetonitrile/0.1% aqueous TFA and equilibrated with 0.1% aqueous TFA. The sample was applied and the run-through and wash fractions were collected in glas vials and dried by vacuum rotation.

### Chemical cleavage of O-glycans

The sample was taken up in 50 µl of 50 mM sodium hydroxide/1 M sodium borohydride and incubated for 16 h at 50 °C. The reaction was stopped by addition of a 50% aqueous solution of acetic acid. Desalting of the sample was performed in a batch procedure with a 50% Dowex 50Wx8(H +) water suspension. Borate in the dried sample was removed by distillation from acidified methanol as borate methylester in a flow of nitrogen at 40 °C. The samples (O-glycan alditols) were desalted and separated from protein/peptide contaminants by solid-phase extraction on graphitized carbon (Extract-Clean columns, Carbograph, 150 mg, 4.0 ml, Alltech, Nettetal, Germany).

### Methylation of glycans and MALDI mass spectrometry

In brief, the methylation of extensively dried samples was performed according to the method by Ciucanu and Kerek based on finely powdered sodium hydroxide in dry DMSO/methyl iodide^56^. Methylated glycans in methanol or peptides in 0.1% aqueous TFA were applied onto a MALDI stainless steel target (Bruker) together with an equal volume of matrix (α-cyano-4-hydroxy-cinnamic acid, saturated solution in 50% acetonitrile/0.1% aqueous TFA). MALDI-MS analysis was performed in the positive ion reflectron mode (HV acceleration 25 kV) on an UltrafleXtreme MALDI-TOF-TOF mass spectrometer (Bruker Daltonics) using the acquisition software FlexControl 3.3 and the data evaluation software FlexAnalysis 3.3 (Bruker Daltonics). Peptide and glycan sequencing was performed by laser-induced dissociation (LID) in the post-source decay (PSD) mode. Mass annotation was performed in FlexAnalysis and peak lists were generated after manual inspection of the entire mass range for correct isotope peak annotation. The molecular masses of the alditols (M + Na) were searched in standard mass lists, and finally identified by MS/MS using the fragmentation annotation tool provided in the GlycoWorkBench platform. Peptide identification was assisted by the Protein Prospector tool MS-digest.

### Characterization of pc-asp anti-asprosin antibody

To test the specificity and sensitivity of pc-asp anti-asprosin antibody, recombinant asprosin**,** N-terminal fibrillin-1 (rF90), C-terminal fibrillin-1 (rF6), human albumin, and human IgG (Sigma-Aldrich, Darmstadt, Germany) were diluted in PBS and coated to the wells of a 96-well ELISA plate in 100 µl/well followed by overnight incubation at 4 °C. After washing with PBS containing 0.05% Tween 20 (PBST), the wells were incubated with 5% milk in PBS for 2 h for blocking. Purified pc-asp anti-asprosin antibody was diluted 1:10,000 in 2.5% milk in PBS and applied to the wells for 1 h at RT. The wells were washed three times with PBST, and mouse anti-rabbit IgG-HRP conjugate (#211–032-171, Jackson ImmunoResearch, Pennsylvania, USA) was then added at dilution 1:2000 in 2.5% milk in PBS and incubated for 30 min at RT. After washing the wells with PBS, the signal was developed by adding 1-Step Ultra TMB-ELISA Substrate Solution (Thermo Fisher Scientific, Massachusetts, United States) followed by an incubation for 1–3 min. The reaction was stopped with an equal volume of 30% H_2_SO_4_ (stopping solution) and the optical density (OD) was measured 450 nm.

### Asprosin sandwich ELISA

Pc-asp anti-asprosin antibody (capture antibody) was coated at 3 µg/ml in antibody coating buffer (50 mM carbonate/bicarbonate buffer, pH 9.6) on 96-well ELISA plates in a volume of 100 µl/well and incubated at 4 °C overnight. Unbound antibodies were washed off with PBST. The plate was incubated with 200 µl of 5% milk in PBS for 2 h. Asprosin standard, human serum, and plasma samples were diluted in PBS. Cell culture media, breast milk, saliva samples, and pull-down fractions were added directly to the ELISA plate. After incubating 100 µl of a serial dilution of asprosin standard and samples at RT for 2 h, the plate was washed for three times with PBST. 100 μl of mab anti-asprosin antibody (detection antibody) were added to the plate at a dilution of 1:2000 (0.5 µg/ml) in 2.5% milk in PBS for 1 h at RT. The plate was washed three times with PBST before adding 100 µl of rabbit anti-mouse IgG-HRP conjugate (secondary antibody) at 1:2000 in 2.5% milk in PBS for 30 min at R.T. After final three washes with PBS, 100 µl of 1-Step Ultra TMB-ELISA Substrate Solution was added and incubated for 15–20 min for color development. The reaction was stopped with an equal volume of 30% H_2_SO_4_ (stopping solution) and the optical density (OD) was read at 450 nm. Inter-assay CV was determined for identical sample duplicates analyzed using two different plates from the same lot number on the same ELISA plate reader. Intra-assay CV was determined for identical sample duplicates on the same plate.

### Western blot

Protein samples were mixed with Laemmli Sample Buffer (Bio-Rad, Hercules, USA) containing 10% (v/v) β-mercaptoethanol and heated to 95 °C for 10 min. Samples were subjected to electrophoresis on 10%, 12%, or 7.5% SDS-PAGE. Proteins were stained using Coomassie Blue Staining. For western blotting, proteins are separated by SDS-PAGE, transferred to PVDF transfer membrane, 0.45 µm (Thermo Fisher Scientific, Massachusetts, United States). Membranes were blocked with Pierce Protein-Free (TBS) Blocking Buffer (Thermo Fisher Scientific, Massachusetts, United States) for one hour, then incubated with the following primary antibodies: pc-asp anti-asprosin antibody, 1:5000 (0.2 µg/ml), mab anti-asprosin antibody (clone Birdy-1), 1:1000 (1 µg/ml), fibrillin-1 (rF90) 1:5000 (0.5 µg/ml), fibrillin-1 rabbit monoclonal antibody (CPTC-FBN1-3) 1:2500 dilution (1 µg/ml). and secondary antibody, mouse anti-rabbit IgG-HRP conjugate or goat anti-mouse IgG-HRP conjugate, 1:5000. All antibodies were diluted in blocking buffer. Signals were developed with SuperSignal West Pico PLUS Chemiluminescent Substrate (Thermo Fisher Scientific, Massachusetts, USA). Blotted PVDF membranes used for Fig. 1F were cut and incubated with indicated primary antibodies.

### Cartilage sample preparation

Tibial plateau was obtained from one patient undergoing routine total knee joint replacement surgery. The resected tissue was incubated in isotonic sodium chloride solution 0.9% and kept for less than 3 h at 4 °C before macroscopic observation. Based on the morphological appearance (color and gloss) of the cartilage surface, a small region of healthy cartilage was selected at the lateral periphery of the plateau and further processed. A piece of cartilage was dissected using a scalpel and embedded in OCT (Thermo Fisher Scientific, Massachusetts, USA), snap frozen and kept in -80 °C freezer until cryosectioning.

### Immunofluorescence and toluidine blue staining

To identify the optimal tissue fixation and treatment for asprosin detection by the pc-asp anti-asprosin antibody, air-dried cartilage cryosections were subjected to one of the following preparations: (1) fixed with -20 °C ice cold acetone or acetone:methanol (1:1) mixture and then incubated at 4 °C for 4 min, dried for 60 min at room temperature and then rehydrated with PBS for 10 min; (2) fixed with 4% paraformaldehyde for 10 min at room temperature and washed three times with PBS for 10 min. Afterwards, sections treated with 0.1% Triton X-100 plus 0.05% Tween-20 in PBS for 15 min. For enzyme digestion, air-dried acetone treated and rehydrated cryosections were directly submitted to further incubation in the corresponding digestion solutions: pepsin digestion: 0.025% pepsin (#516,360, pepsin from porcine stomach mucosa, Sigma-Aldrich, Darmstadt, Germany) in 0.2 N HCl for 10 min at 37 °C; hyaluronidase digestion: 1 mg/ml hyaluronidase (#H3506, hyaluronidase from bovine testes, Sigma-Aldrich, Darmstadt, Germany) in digestion buffer (0.1 M sodium dihydrogen phosphate (NaH_2_PO_4_), 0.1 M sodium acetate (NaOAc, pH 5.0) for 10 min at 37 °C; proteinase K digestion: 10 µg/ml proteinase K (#EO0491, Thermo Fisher Scientific, Massachusetts, USA) in 50 mM Tris–HCl pH 7.5 for 1 h at 55 °C; collagenase type I digestion: 1 mg (125 Unit) of collagenase I enzyme (#LS004194, CellSystems GmbH, Troisdorf, Germany) reconstituted in 1 ml HSBS buffer (#14,025,092, Thermo Fisher Scientific, Massachusetts, USA), diluted at 1:100 in HBSS buffer, and incubated with the sections for 30 min at 37 °C. After digestion, sections were rinsed for 3 min with PBST (PBS with 0.05% Tween 20), then incubated with 0.2% Triton X-100 in PBST for 15 min. All specimens were then proceeded identical: incubated in blocking solution (5% normal donkey serum (Dako) containing 0.05% Tween 20 in PBS for 1 h at room temperature and then incubated with pc-asp anti-asprosin antibody at a 1:70 dilution in Antibody Dilution Buffer (AKV-Puffer) (Fa. DCS # AL120R100, Germany) containing 1 µg/mL DAPI (Thermo Fisher Scientific, Massachusetts, USA) for 1 h at room temperature, followed by two washing steps with PBST for 5 min. Slides were incubated with secondary antibody donkey anti-rabbit Alexa 568 (#A10042, Invitrogen, Thermo Fisher Scientific, Massachusetts, USA) diluted 1:300 in AKV-Puffer for 1 h in the dark at room temperature, followed by two washes with PBST for 1 min and a final PBS wash for 1 min. Slides were coverslipped with ProLong Gold Antifade Mountant (#P36934, Thermo Fisher Scientific, Massachusetts, USA).

For immunofluorescence chondrocyte cultures, chondrocytes were seeded on uncoated glass coverslips at a density of 1 × 10^5^ cells per well in 12-well plates. Cells were kept in culture for 12 days, then washed with PBS for 5 min, fixed with ice cold acetone:methanol (1:1) for 10 min at − 20 °C, blocked with 1% bovine serum albumin (#11,930.03, SERVA Electrophoresis GmbH, Heidelberg, Germany) in PBS, and then incubated with pc-asp anti-asprosin antibody (1:70) in 0.5% BSA in PBS for 1 h at room temperature. The coverslips were washed two times with PBST for 5 min and incubated with secondary antibody goat anti-rabbit Alexa 555 (# A27039, Invitrogen, Thermo Fisher Scientific, Massachusetts, USA) for 30 min in dark at room temperature, then washed two times with PBST for 1 min and one time with PBS for 1 min. Subsequently, coverslips were air-dried for 10 min and mounted to the slide using ProLong Gold Antifade Mountant with DAPI. Confocal images were obtained using a Leica SP8 confocal microscope (Leica Camera AG, Germany) and Leica LAS AF Lite 4.0 software. Images were further processed using Fiji/ImageJ software to obtain average Intensity Z-projection.

Cartilage cryosections with 10 µm thickness were stained with Toluidine Blue O (#1B481, Chroma-Gesellschaft Schmid GmbH & Co., Köngen, Germany) according to the following procedures: sections were dried for 1 h at 37 °C, washed in distilled water for three times, stained in Toluidine Blue O working solution for 2 min, washed in distilled water for 3 times, dehydrated quickly in 95% ethanol and then in 100% ethanol for two times, washed in xylol two times for 3 min, and coverslipped with EUKITT UV O. Kindler EUKITT UV mounting media (ORSAtec GmbH, Germany). Toluidine-stained cartilage sections were imaged with Zeiss AX10 light microscope equipped with Zeiss plan-Neofluar 10 × /0.3 objective, Hitachi HV-F202 camera and Diskus software (version number 5.0.6353 #7599).

### Surface plasmon resonance

SPR experiments were performed as described previously^57^ using a BIAcore 2000 system (BIAcore AB, Uppsala, Sweden). Pc-asp anti-asprosin antibody was immobilized at 8000 RUs to a CM5 sensor chip using the amine coupling kit following the manufacturer’s instructions (Cytiva, Uppsala, Sweden). Interaction studies were performed by injecting 0–80 nM recombinant asprosin and RPE supernatant pull-down fractions in HBS-EP buffer (0.01 M HEPES, pH 7.4, 0.15 M NaCl, 3 mM EDTA, 0.005% (v/v) surfactant P20) (Cytiva, Uppsala, Sweden). Human asprosin was immobilized at 500 RUs to a CM5 sensor chip using the amine coupling kit. Interaction studies were performed by injecting 0–40 nM pc-asp, mab and Strep-ab in HBS-EP buffer. Kinetic constants were calculated by nonlinear fitting (1:1 interaction model with mass transfer) to the association and dissociation curves according to the manufacturer's instructions (BIAevaluation version 3.0 software). Apparent equilibrium dissociation constants (K_D_ values) were then calculated as the ratio of k_d_/k_a_.

### Exercise study

Fifteen male healthy participants, age = 27.5 ± 3.1 years, weight = 78 ± 7.7 kg, height = 1.81 ± 0.05 m, BMI = 23.9 ± 1.9 kg/m^2^ were recruited by German Sport University Cologne, Cologne, Germany to study the effect of exercise on the serum asprosin level (Supplementary Table S7). Inclusion criteria were age from 20 to 35 years and physical fitness. Exclusion criteria were musculoskeletal disorder, acute and chronic injuries, trauma or surgery of lower extremities’ joints. Subjects were asked not to exercise 24 h prior to the study and to come to the institute either by car or public transportation. The subjects remained seated for 30 min before the first blood sampling (t0). Further blood samples were drawn immediately afterwards (t1), and then 30 min (t2), 60 min (t3), and 120 min (t4) after running. Blood (5.0–7.5 ml) was collected by venipuncture using serum-gel monovettes1 (Sarstedt, Nümbrecht, Germany). After blood coagulation (30 min, RT), serum was isolated by centrifugation (10 min, 3000 rpm) and stored at − 80 °C.

### Osteoarthritis patients study

Fourteen patients diagnosed with unilateral and bilateral symptomatic hip OA, validated by conventional anterior/posterior and lateral pelvic radiographs (7 females, 7 males, age = 61.4 ± 10.5 years, height = 1.74 ± 0.07 m, weight = 79.7 ± 16.4 kg, BMI = 26.2 ± 4.7 kg/m^2^) at the Department of Orthopaedics at the University Hospital of the Goethe University Frankfurt in Germany voluntarily participated in the study. All patients reported pain for the ipsilateral hip although, the contralateral hip was free from any symptoms before the surgery. At the time of all follow-up measurements all patients were pain-free. The period of prospective examination began before surgery and ended nearly 1 year after initial THR. The patients were recruited and examined as previously reported^21^. Anthropometric measures of the two analyzed cohorts in this study are shown in Supplementary Table S7.

### Blood and saliva samples

Fasted blood (3–5 ml) and saliva samples (1–2 ml) were collected 8–9 h after overnight fasting and for non-fasted samples, they were collected 3 h after lunch meal. Serum separator tube (SST) was used to separate serum from the whole blood specimen. Samples were allowed to clot for 30 min before centrifugation for at least 15 min at 1000 × g. serum was removed, aliquoted and kept frozen at − 80 °C. For saliva samples, the required amount of saliva samples was collected, and subjects were asked to refrain from eating, drinking, smoking, chewing gum, brushing their teeth, or using mouthwash for at least 30 min before providing their samples. Samples were centrifuged for 5 min at 1000 × g and aliquoted and kept frozen at − 80 °C.

### Statistical analysis

Data are expressed as mean ± SD. Statistical analyses were performed using GraphPad Prism software version 8.0.2 and the significance of differences between groups was determined by applying an unpaired two-tailed Student’s test. Values of *P* ≤ 0.05 were considered significant. For the exercise study, one-way analysis of variance (ANOVA) with repeated measures and Dunnett's multiple comparisons test for post hoc analysis were performed to detect significant differences in asprosin levels between time points. The significance level was set at *P* ≤ 0.05.

## Supplementary Information

Supplementary Information.
