# Supplementary material for: Sensitive asprosin detection in clinical samples reveals serum/saliva correlation and indicates cartilage as source for serum asprosin
Source: Sci Rep. 2022 Jan 25;12:1340. doi: 10.1038/s41598-022-05060-x (PMC8789892; doi:10.1038/s41598-022-05060-x)

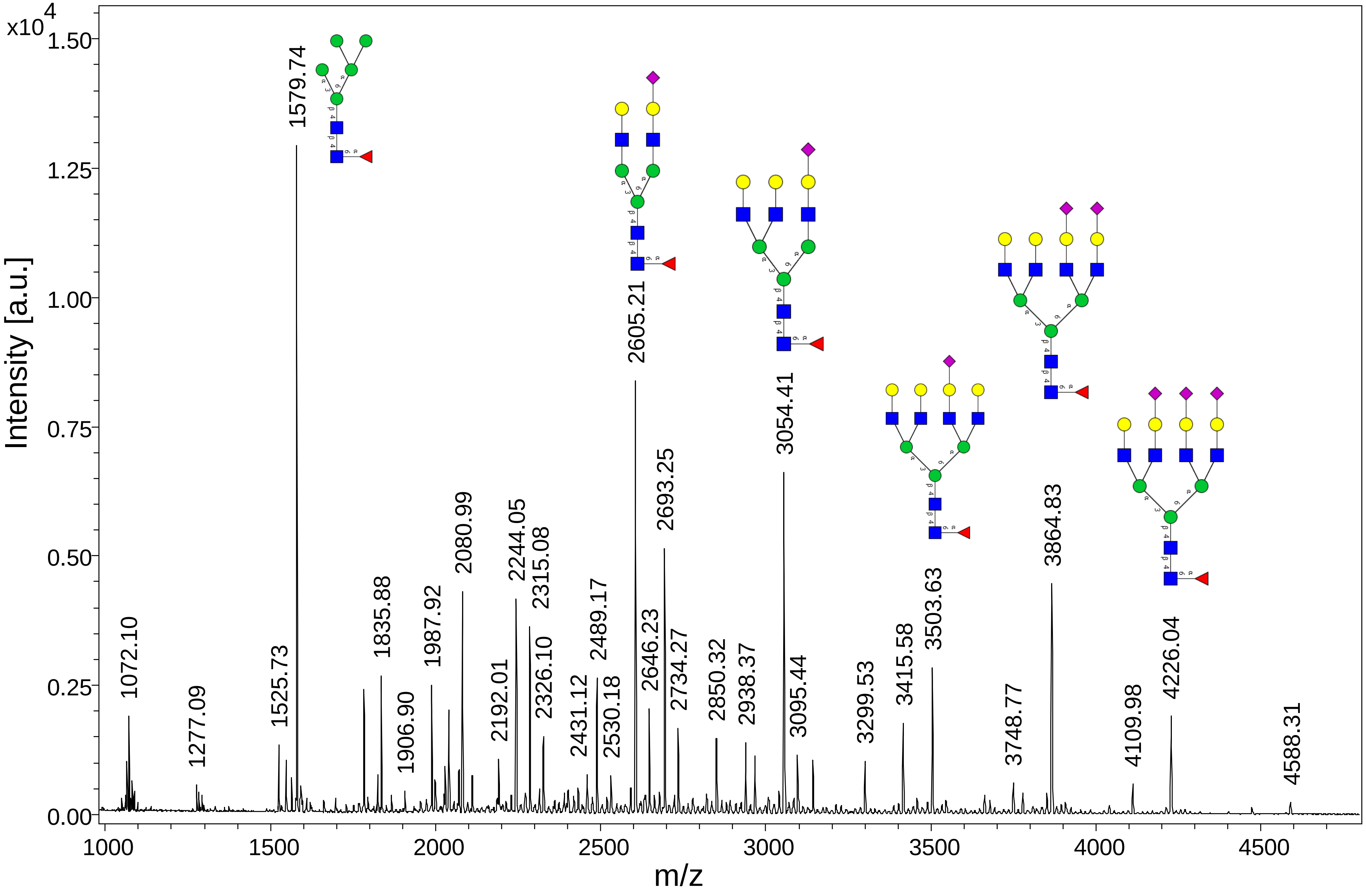

Supplementary Figure S1: Survey MALDI mass spectrum of permethylated N-glycans from recombinant asprosin. PNGaseF-liberated N-glycans were methylated and analyzed by positive ion reflectron MALDI mass spectrometry. The profile is characterized by dominant high-mannose N-glycan M5 and core-fucosylated complex-type N-glycans with two to four antennae. Besides fully processed species, the profile contains significant amounts of partially processed (truncated) –Gal species.

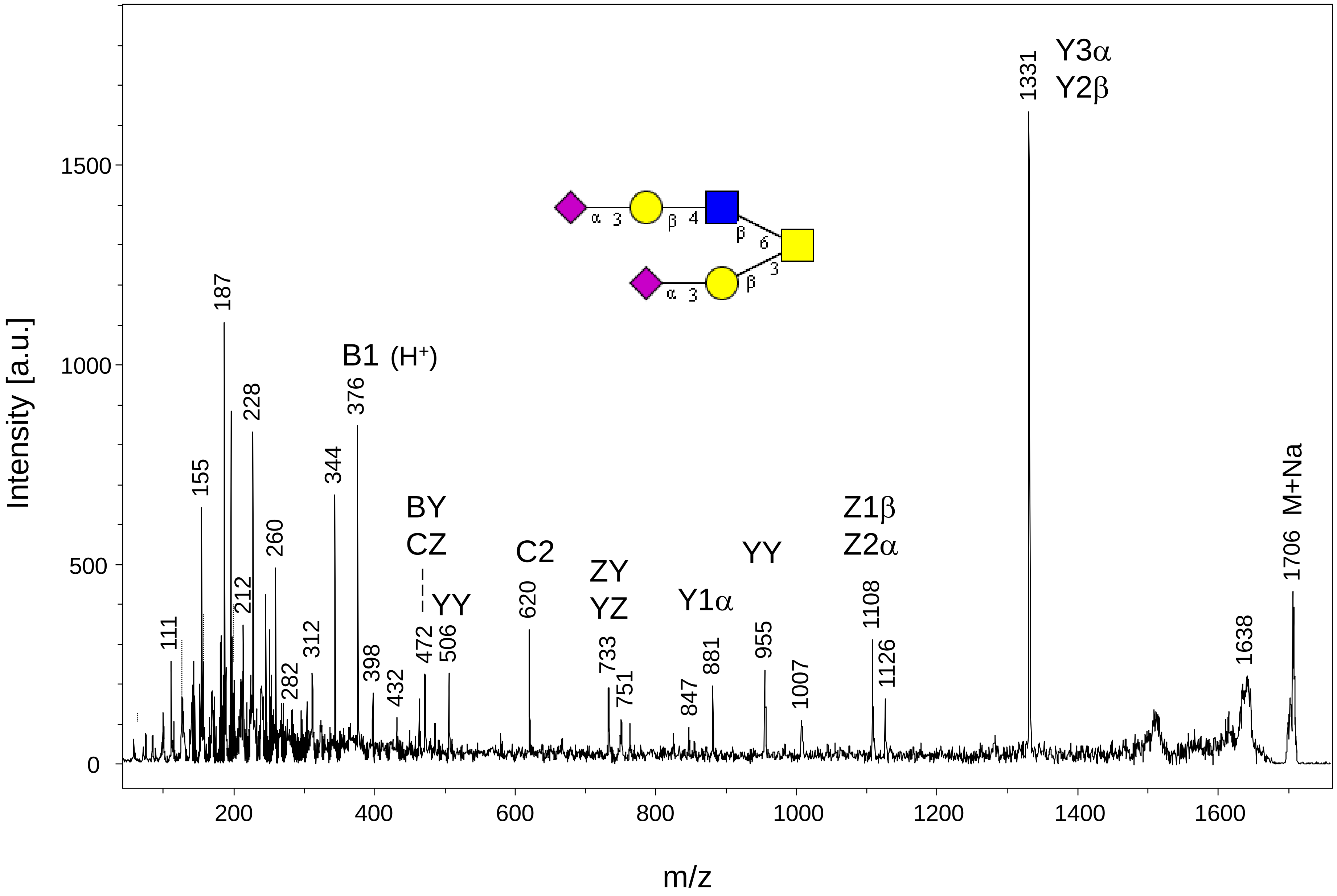

Supplementary Figure S2: Post-Source-Decay MALDI mass spectrum of permethylated O-glycan alditol detected at precursor ion mass m/z 1706. Only one major precursor ion was detectable in MALDI survey spectra of O-glycan alditols derived from recombinant asprosin. PSD analysis of the compound detected at m/z 1706 revealed a fragment pattern supporting a hexasaccharide (disialylated core 2 tetrasaccharide) of the structure shown.

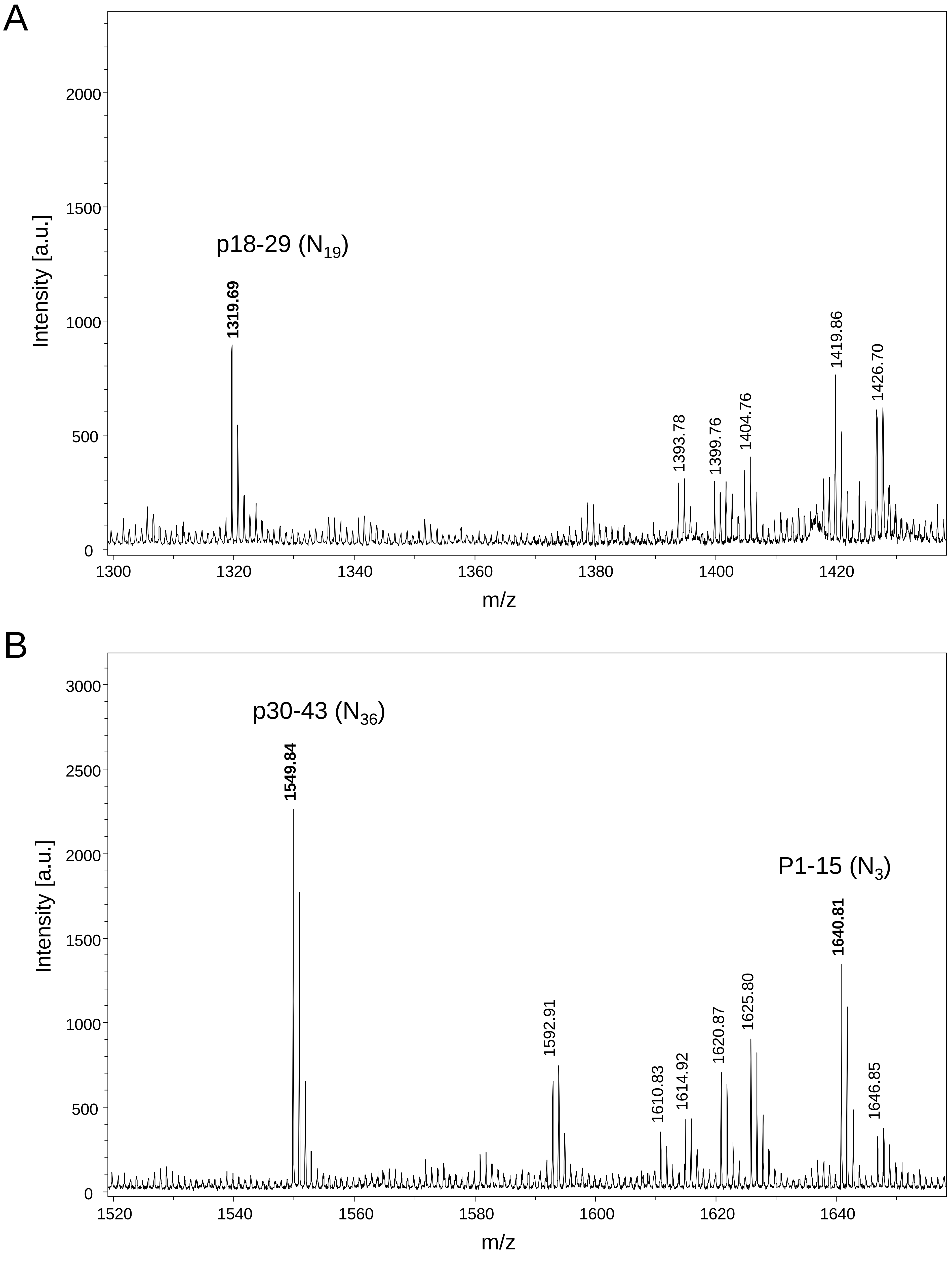

Supplementary Figure S3: MALDI mass spectra of de-N-glycosylated peptides from recombinant asprosin after double digestion with V8 and trypsin. A. The section shows a mass range of the spectrum from m/z 1300 to 1430 to highlight the signal at the monoisotopic mass m/z 1319.69, which corresponds to the native peptide mass m/z 1318.66 of p18-29 (A**N**VSLASWDVEK). The observed mass shift of +1 results from conversion of Asn to Asp during enzymatic liberation of N-linked glycans. **B**.The section shows a mass range of the spectrum from m/z 1520 to 1650 to highlight the signals at the monoisotopic masses m/z 1549.84 and 1640.81, which correspond to the native peptide masses m/z 1548.82 of p30-43 (TAIFAFNISHVSNK) and m/z 1639.66 of p1-15 (STNETDASNIEDQSE). The observed mass shift of +1 results from conversion of Asn to Asp during enzymatic liberation of N-linked glycans.

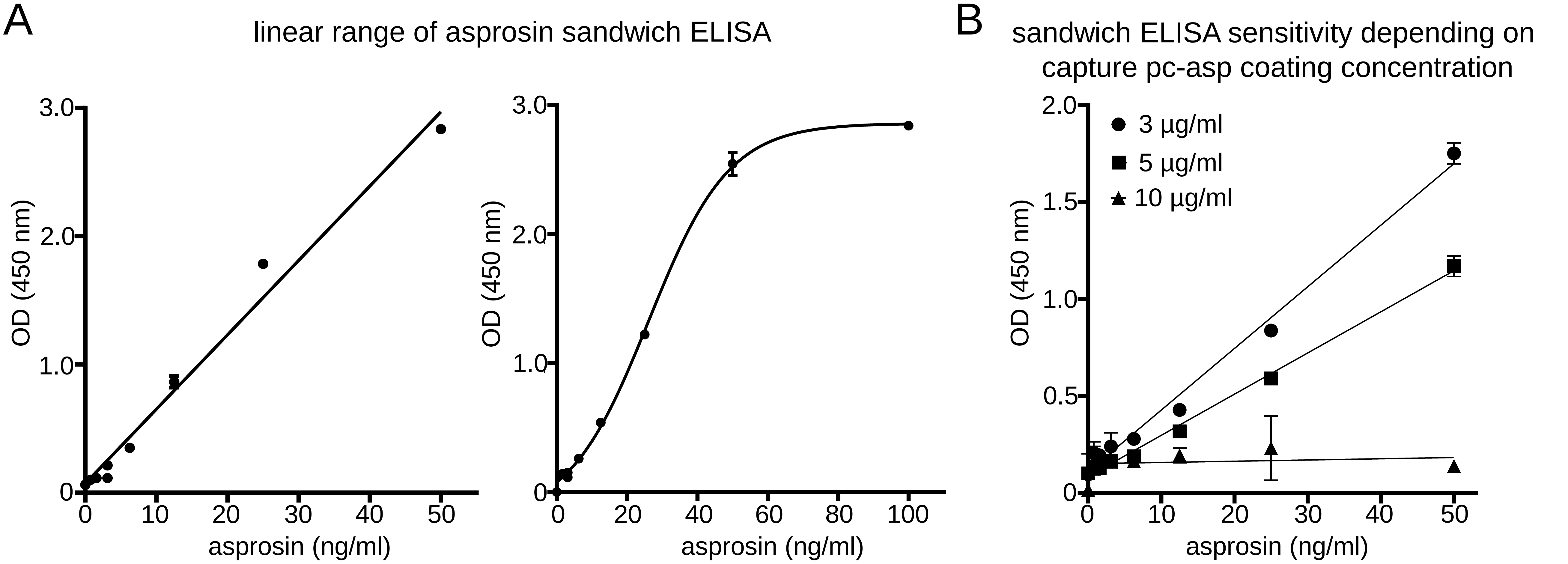

Supplementary Figure S4: Critical parameters of established asprosin sandwich ELISA. A. Linear range of asprosin sandwich ELISA was determined to be between 0 – 50 ng/ml. **B.** Concentration of coated capture antibody (pc-asp) affects sensitivity of asprosin sandwich ELISA. Data were analyzed using Graphpad Prism version 8.0.2.

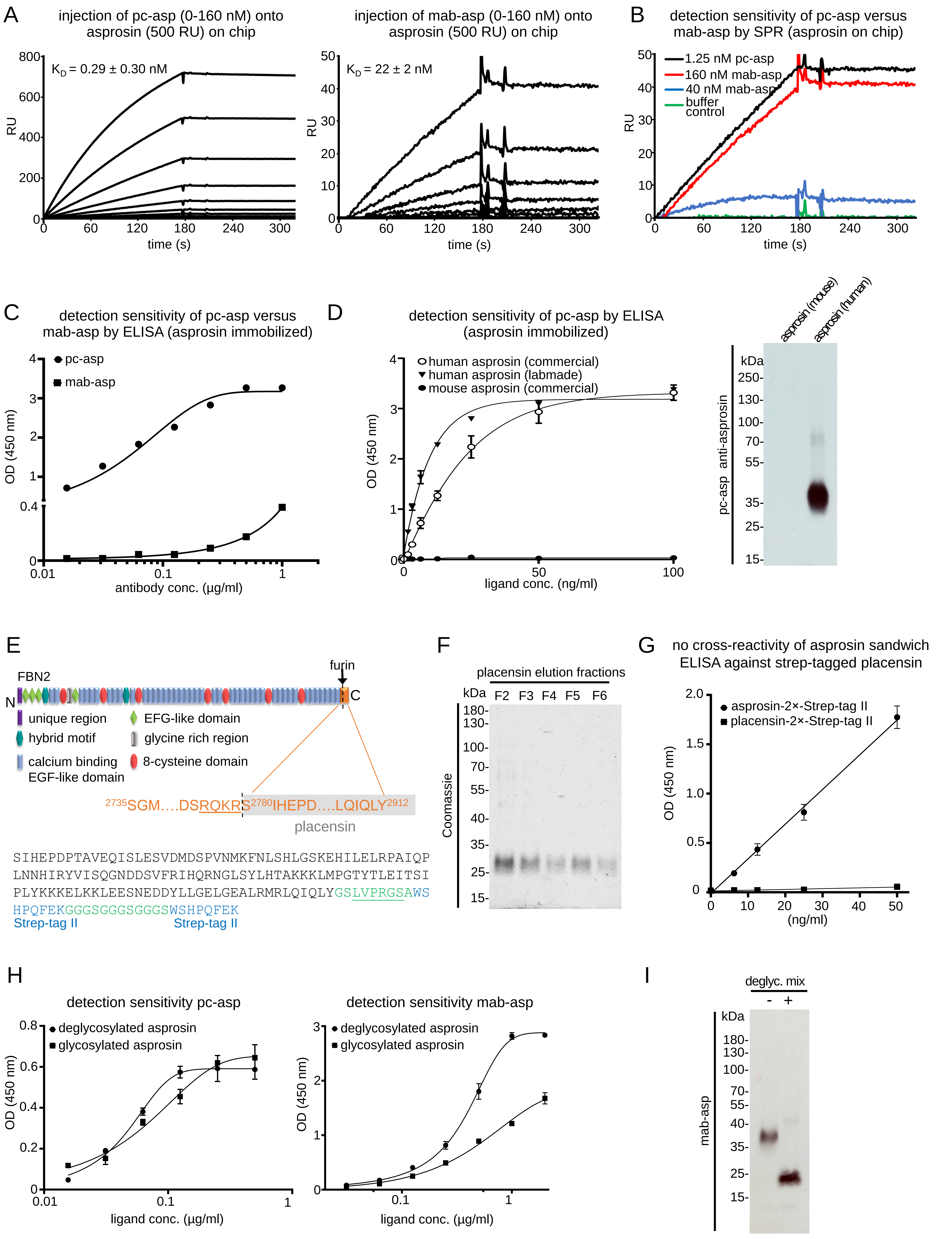

Supplementary Figure S5: Affinity of pc-asp versus mab against glycosylated and deglycosylated asprosin. A. Measurement of pc-asp and mab-asp affinity by SPR. Sensorgrams of injections of 2-fold serial dilutions (0-160 nM) of (left) pc-asp and (right) mab antibodies onto asprosin immobilized on chip. The calculated affinity (K_D) of pc-asp was 0.29 ± 0.3 nM and mab was 22 ± 2 nM. **B.** Sensorgrams showing higher sensitivity of pc-asp (1.25 nM) compared to mab (160 nM) to immobilized asprosin on chip. **C.** pc-asp shows a higher sensitivity against immobilized human asprosin (purchased from Biolegend, #761902) versus mab. **D.** (left) pc-asp antibody showed high specificity to human asprosin and no cross reactivity to mouse asprosin in direct ELISA assay and western blot analysis (right). **E.** (top) Domain structure of fibrillin-2 and and sequence of its C-terminal propeptide, placensin (marked in grey). Placensin consists of 133 amino acids (S^{2780}-Y^{2912}) after furin cleavage from pro-fibrillin-2 (position of furin cleavage site within domain structure is marked by arrow, furin cleavage site is underlined). (bottom) Placensin sequence with a C-terminally placed double Strep-tag II which was overexpressed in HEK293 cells. Residues representing linker regions are indicated in green, thrombin cleavage (LVPRGS) site is underlined, and Strep-tag II sequences are marked in blue. **F.** Coomasie stained quality control gel of eluted fractions (F2-F6) of human placensin after affinity chromatography. **G.** Specific detection of recombinantly expressed asprosin containing a C-terminally placed double Strep-tag II by asprosin sandwich ELISA. No crossreactivity to recombinantly expressed double strep-tagged placensin was observed by asprosin sandwich ELISA. **H.** Sensitivity of pc-asp and mab to glycosylated and deglycosylated asprosin using direct ELISA. (left) Pc-asp antibody shows almost equal sensitivity to immobilized glycosylated and deglycosylated asprosin. (right) mab-asp antibody shows low sensitivity to glycosylated compared to deglycosylated asprosin. **I.** Western blot with mab anti-asprosin detecting glycosylated (≈ 37 kDa) and deglycosylated asprosin (≈25 kDa). (Deglycosylation was performed with Protein Deglycosylation Mix II, P6044S, NEB, Germany). ELISA data were analyzed using Graphpad Prism version 8.0.2. Association and dissociation rates were obtained using BIAevaluation version 3.0 software. Apparent equilibrium dissociation constants (KD values) were then calculated as the ratio of kd/ ka.

A

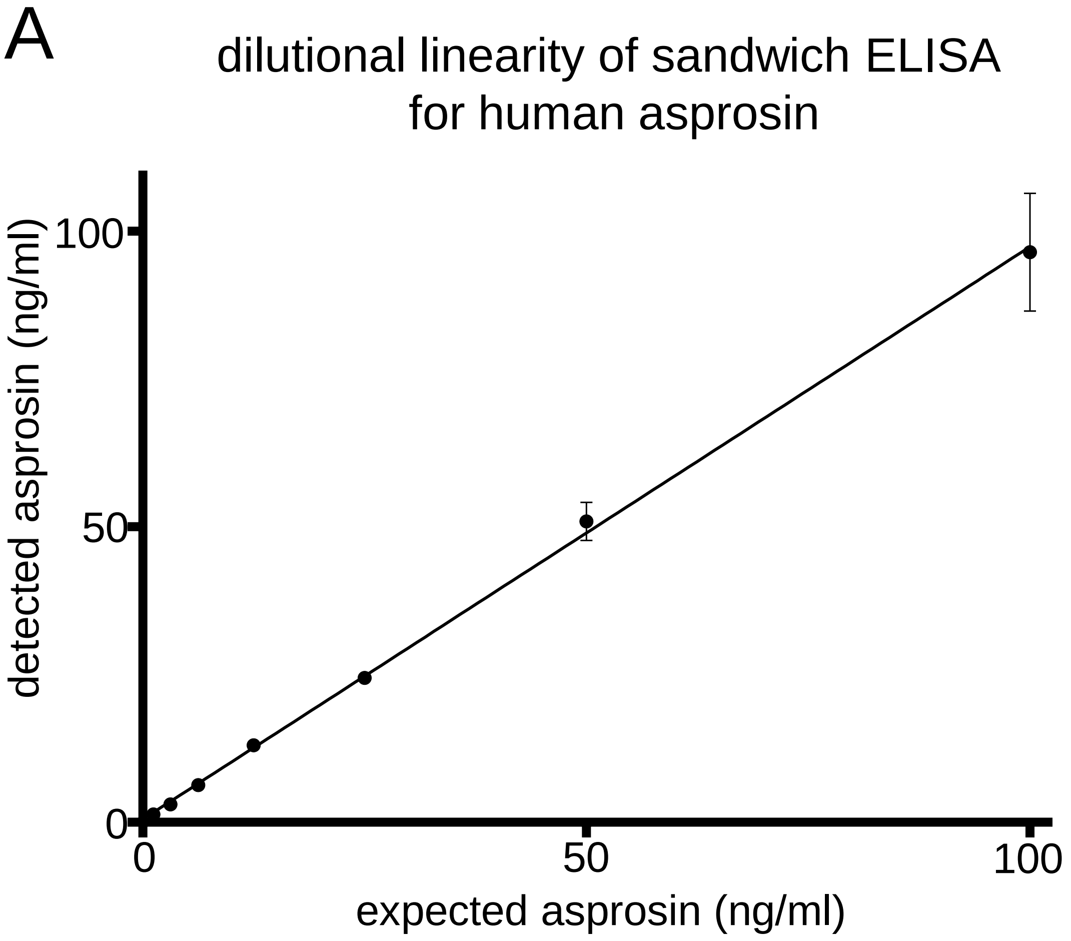

B

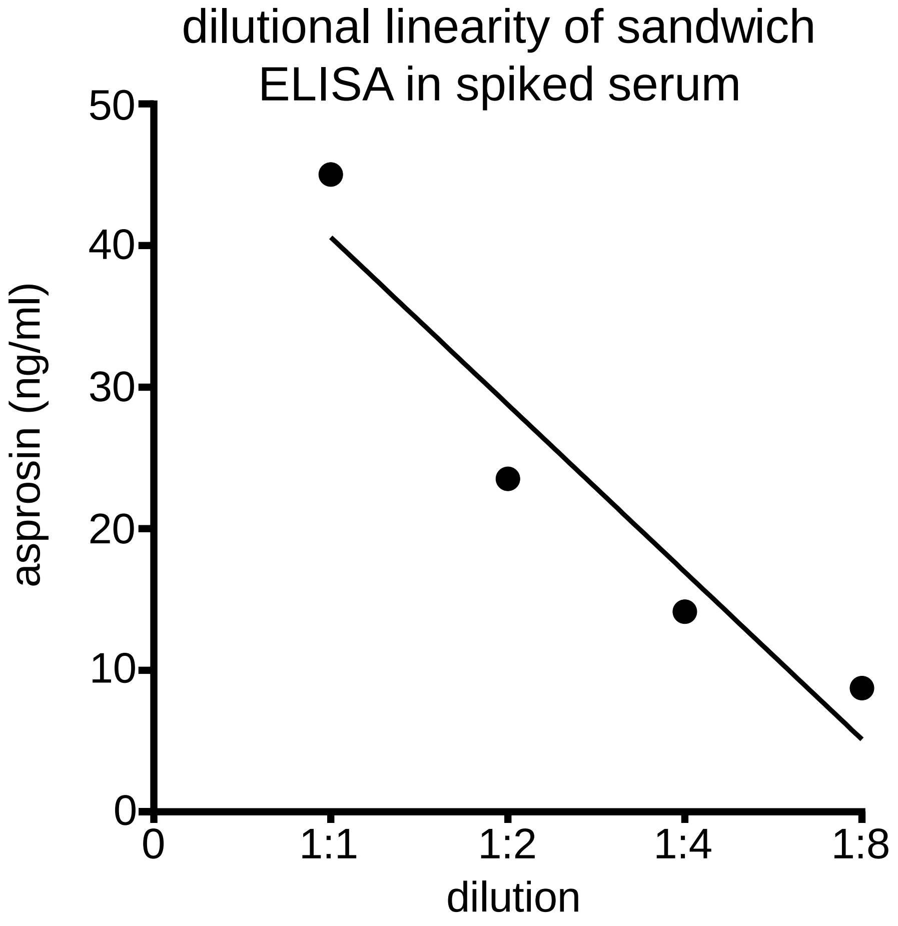

dilution factor	expected (ng/ml)	detected (ng/ml)	recovery %
1	50	45.02	90.04
2	25	23.52	94.08
4	12.5	14.14	113.12
8	6.25	7.14	114.34

C

spike and recovery assessment of asprosin sandwich ELISA

	sample	expected (ng/ml)	detected (ng/ml)	recovery (%)
plasma	1	30	23.18	77.28
	2	30	27.34	91.16
	3	30	30.63	102.11
	4	30	28.36	94.55
	5	30	26.24	87.48
serum	1	30	28.31	94.39
	2	30	32.37	107.91
	3	30	25.38	84.63
	4	30	32.22	107.41
	5	30	36.33	121.10

expected (ng/ml)	detected (ng/ml)	recovery (%)
50	59.99	119.98
	45.02	90.04
	40.30	80.61
25	29.21	116.86
	24.08	96.34
	24.56	98.26

Supplementary Figure S6: Linearity of dilution and spike and recovery assessment of asprosin sandwich ELISA. A. Dilution of asprosin in DMEM, 10 % FCS does not affect accuracy and precision of asprosin detection, correlation of detected asprosin and excepted asprosin concentrations using Spearman correlation = 0.9996. **B.** (left) Dilution of asprosin spiked serum with PBS does not affect the accuracy and precision of asprosin detection, the average recovery is 102.8 %. (right) Table showing expected and detected asprosin concentration values, in addition to estimated asprosin recovery. **C.** (left) Investigation of matrix effect on asprosin detection by using spiking asprosin in various serum and plasma samples, showing average recovery 96.8%. (right) Assessment of spiked asprosin recovery in serum samples before and after 1:2 dilution. Data were analyzed using Graphpad Prism version 8.0.2.

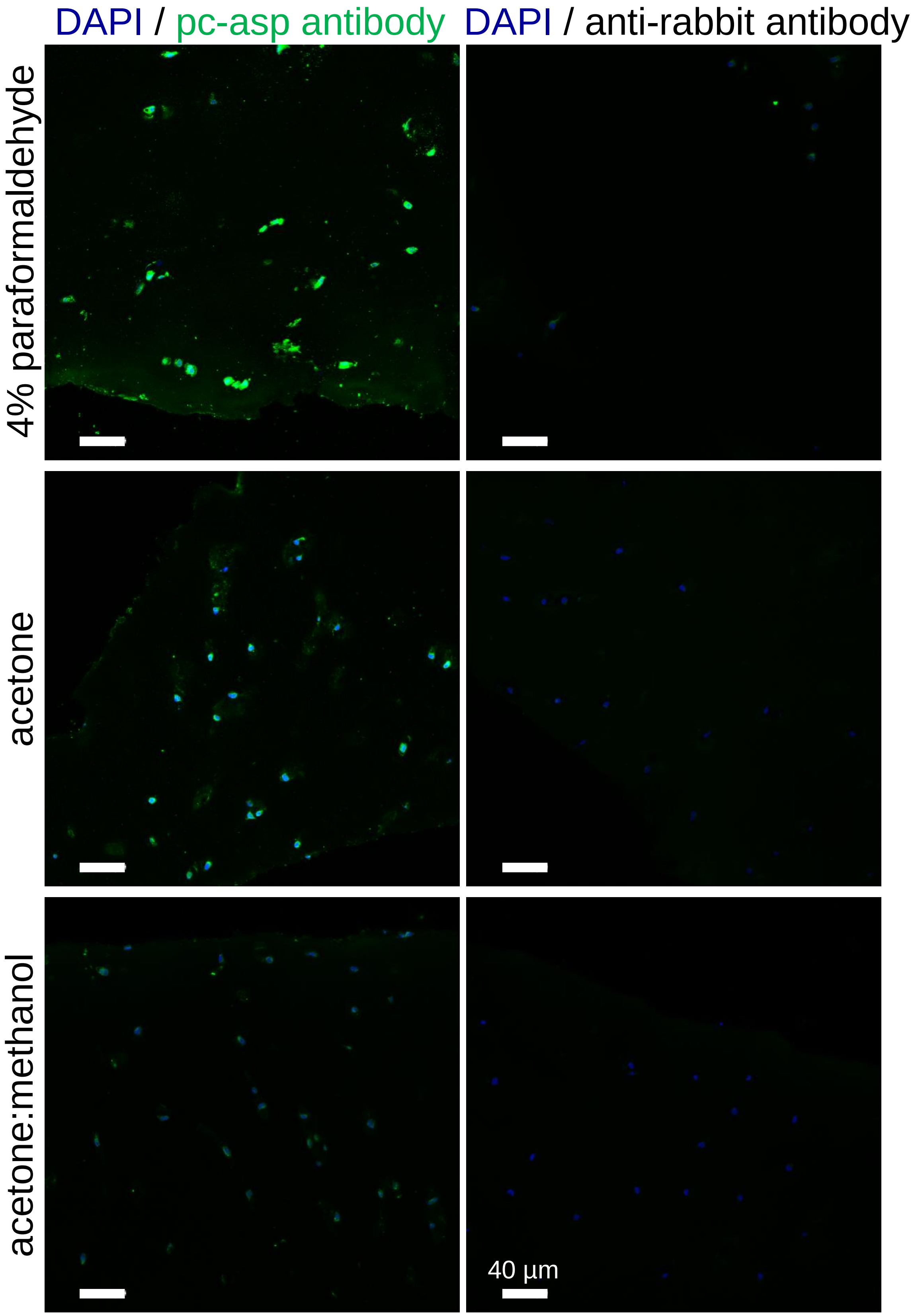

Supplementary Figure S7: Effect of fixation and treatments on asprosin recognition by immunostaining in human cartilage.

Immunodetection of asprosin in human chondrocytes *in situ*. Cryosections from cartilage specimen (top raw) were fixed with 4% paraformaldehyde, (middle raw) treated with acetone, or (bottom raw) fixed with acetone:methanol mixture. The left images showed sections incubated with pc-asp asprosin antibody (green) and DAPI (blue). The right images showed sections incubated only with secondary antibody (control) and DAPI. Confocal images were obtained from a Leica SP8 confocal microscope and Leica LAS AF Lite 4.0 software. Images were further processed using Fiji/ImageJ software to obtain average intensity Z-projection.

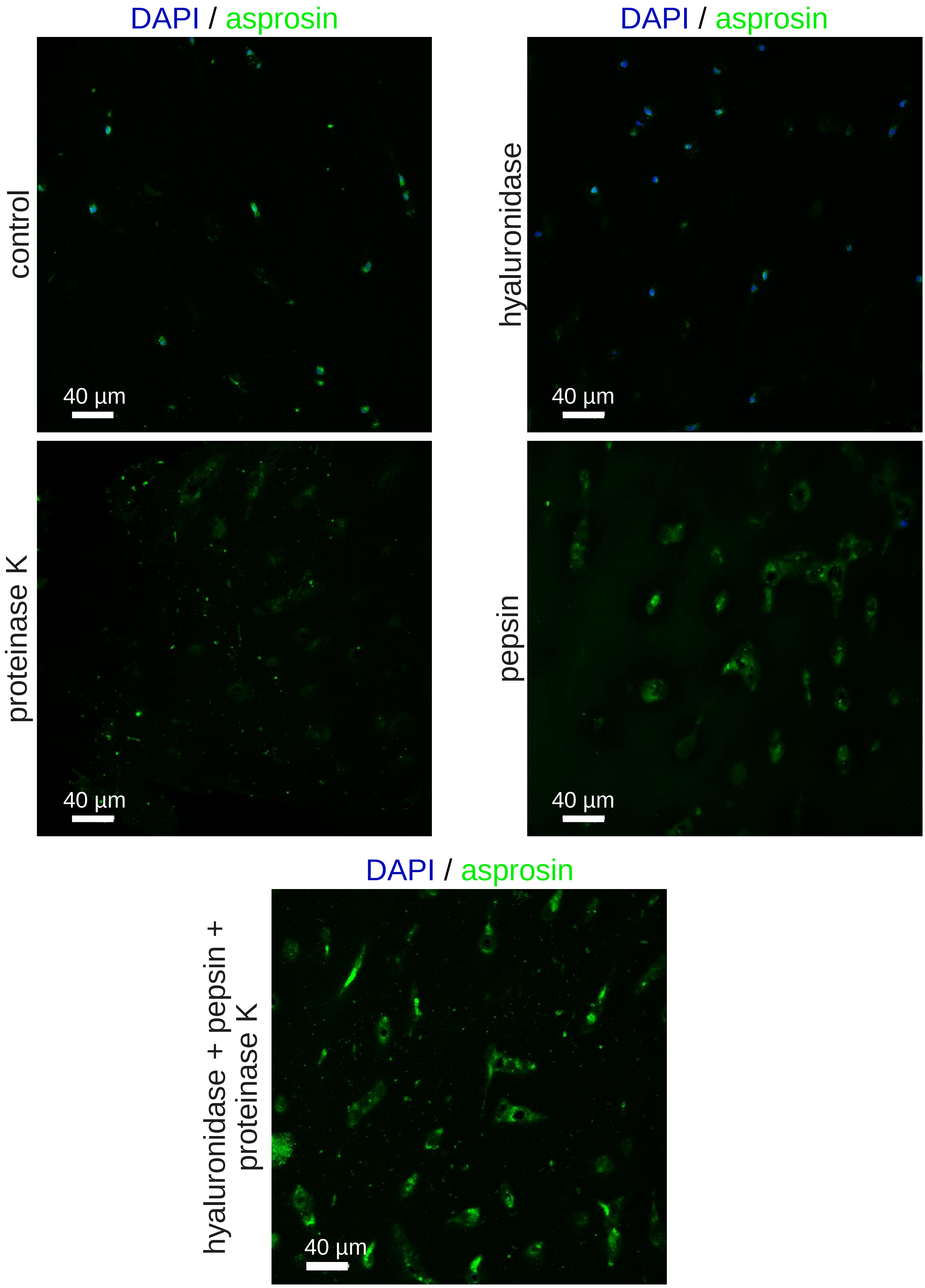

Supplementary Figure S8: Effect of digestion by various enzymes on asprosin detection by immunostaining in human cartilage.
Cartilage cryosections were treated (top, left) with acetone only, or additionally (top, right) digested with hyaluronidase, or (middle, left) with proteinase K, or (middle, right) with pepsin, or (bottom) with a mixture of all of these enzymes together. The signal of pc-asp asprosin was detected in green, DAPI nuclei staining in blue. Confocal images were obtained from a Leica SP8 confocal microscope and Leica LAS AF Lite 4.0 software. Images were further processed using Fiji/ImageJ software to obtain average Intensity Z-projection.

Supplementary Table S1: Reported asprosin concentrations in clinical samples. Shown data are from studies available on PubMed, search term “asprosin” until June 2nd, 2021.

Year	first author	PMID	range of detected asprosin	amounts (ng/ml)	sample	BMI	age (years)	total number, sex (f/m)	asprosin detection kit	detection range	minimal amounts detectable	intra-assay CV	inter-assay CV
2016	Romere	27087445	5-12 nM (nonfasted- fasted); (nonobese- obese)	185-444	plasma	no information given	no information given	23,	capture: mouse mab anti-asprosin against aa 106–134 (human profibrillin aa 2838–2865), detector: polyclonal goat anti-asprosin aa 6–19 (human profibrillin aa 2737–2750) by Abnova				
2017	Zhang	29104036	ctrls: 1.77 (1.24-3.45) ng/ml; diabetes type 2: 3.52 (1.50-7.17) ng/ml	1.77-7.17	serum	ctrls: 24.81 ± 3.91; T2DM: 25.25 ± 4.20	47.60 ± 7.9549.93 ± 10.99	170, 69/101	Human Asprosin ELISA Kit. Catalogue No: abx257694, Abbexa Ltd, Cambridge, UK		<0.938 ng/ml	<10%.	<6%
2018	Acara	29274804	UAP: admission 7.84 ± 6.57 ng/ml; after 24h angiography: 9.21 ± 12.7 ng/ml	1.27-21.91	serum	no information	60.27 ± 10.67	22, 7/15	Human Asprosin ELISA Kit. Catalogue No: abx257694, Abbexa Ltd, Cambridge, UK				
2018	Wang	29743813	ctrls: 16.22 ± 9.27 ng/mL; impaired glucose regulation: 82.40 ± 91.06 ng/mL; nTD2: 73.25 ± 91.69 ng/ml	6.95-173.46	plasma	ctrls: 22.76 ± 3.61, IGR: 23.86 ± 3.08, nT2DM: 24.73 ± 3.55	63.62 - 66.63	143, 84/59	Human ELISA kit, Wuhan EIAab Science Co. Ltd., China			<10%	<12%
2018	Li	30524197	ctrls: 2-12; TD2: 5-25, PCOS: 7-17 ng/ml	2.0-17.0	plasma	ctrl: 22.68 ± 4.00, T2D: 24.98 ± 3.31, PCOS: 26.68 ± 5.66	ctrls: 37.02 ± 8.16, T2DM: 47.02 ± 4.92, PCOS: 22.68 ± 5.66	160, 160/0, ctrls:66, T2DM: 53, PCOS: 41	Human Asprosin ELISA Kit. Catalogue No: abx257694, Abbexa Ltd, Cambridge, UK	1.563 -100 ng/mL	<0.938 ng/mL	<8%	<10%
2018	Wiecek	30618797	3.7 ± 0.7 nM (f) vs 6.33 ± 3.45 (m) nM	106-362	plasma	23.71 ± 1.58	21.64 ± 1.22 and 22.64 ± 1.49	20, 10/10	SK00229-06 (Aviscera Bioscience, Inc., United States)	37 - 1197 ng/ml (1–32 nmol/L)		<8%	<12%
2019	Alan	30325247	ctrls: 3.69 ± 1.22 ng/ml; PCOS: 6.41 ± 1.89 ng/ml	2.47-8.3	serum	ctrls: 26.64 ± 4.55, PCOS: 26.47 ± 4.44	ctrls: 30.10 ± 6.69, PCOS: 30.30 ± 6.78	156, 156/0	Human Asprosin ELISA Kit. Catalogue No: abx257694, Abbexa Ltd, Cambridge, UK	0.156 - 10 ng/ml		<6%	<8%
2019	Wang	30459402	ctrls: 307 ± 832 ng/ml; obese: 2360 ± 5094 ng/ml	307-7454	serum	25.3 ± 3.6 vs. 41.6 ± 6.3	ctrls: 18–71 obese: 20–63	174, 107/67, ctrls: 57, obese: 117	capture: rabbit polyclonal antibody against asprosin aa 106-134 (Abcam, USA), detector: goat polyclonal antibody against asprosin aa 6–19 (Abnova, Taiwan)				
2019	Chang	31015585	ctrls: 61.5 ± 7.09 ng/ml; PCOS with BMI>25: 71.04 ± 7.81 ng/ml	54.41-78.85	serum	ctrl: 22.59 ± 0.36, PCOS: 25.23 ± 0.28	ctrls: 27.42 ± 0.37, PCOS: 25 ± 0.22	600, 600/0, ctrls: 156, PCOS:444	EIAab, Phoenix Pharmaceuticals, and Millipore Corporation				
2019	Ugur	31049060	ctrls: 14.0 ± 3.75 ng/ml; overweight-obese: 60-130 ng/ml	10.25-130	serum, saliva	ctrl: 21.86 ± 1.96, underweight: 15.5 ± 0.71, overweight: 27.68 ± 1.2	25-44	116	Shanghai sunredbio (SRB) Technology Co. Ltd, catalog no. 201-12-3287, Shanghai, China	1-300 ng/mL	0.756 ng/mL	<10%	<12%
2019	Long	31212299	ctrls: 12.33 ± 4.18 ng/ml, obese: 9.24 ± 4.11 ng/ml	4.03-14.39	plasma	ctrls: boys: 16.17±2.34, girls: 15.36±1.61; obese: boys: 24.77±2.33, girls: 20.80±4.99	8.0-11.0	87, 38/49	Human ELISA Kit; Wuhan EIAab Science Co. Ltd., China)			<12%	<10%
2019	Baykus	31400492	ctrls: 15.9-16.4 ng/ml, study group: 11.7-42.8 ng/ml	11.2-42.8	serum	29.1-32.1	28.1-30.9	179, 179/0	SUNRED BIOSCIENCE, catalogue #:201-12-5592, Shanghai,CHINA)	0.25 - 70 ng/ml	0.214 ng/ml	<8%	<12 %
2019	Wiecek	31510055	ctrls: 4.26 ± 2.05 nM; methabolic syndrome: 4.77 ± 5.17 nM	82-372	serum	25.15–29.23	55 - 70	37, 37/0	SK00229-09 (Aviscera Bioscience, Inc., Santa Clara, CA, USA	37 - 1197 ng/ml (1 - 32 nmol/L)			
2019	Groener	31536600	T1D with and without hypoglycaemia unawareness: 60-280 ng/ml	60-280	plasma	24.4 (20.1–31.3) and 25.4 (21–38.9)	29-75	15, 7/8	Human Asprosin (ASPRO) ELISA Kit, Wuhan Abebio Science Co., Ltd, Wuhan, China, Code: AE26043HU)				
2019	Wang	31775140	ctrls: 0.96 ± 0.48 ng/ml; obese: 1.51 ± 0.44 ng/ml	0.48-1.9	serum	ctrls: 15.67±2.30 vs.obese: 27.44±3.93	8.6-13.1	119, 43/76	USCN Life Science Inc., Wuhan, China			<10%	<10%
2020	Ke	33414826	ctrls: 2.71 ± 0.86 ng/ml; agromegaly patients: 2.18 ± 0.86 ng/ml	2.18-2.71	serum	ctrls: 26.44 ± 3.41, agromegaly: 25.79 ± 3.52	ctrls: 44.5 ± 13.0, agromegaly: 41.3 ± 15.0	189, agromegaly: 39/29, ctrls: 78/43	USCN Life Science Inc., Wuhan, China, Article no. SEA332Hu			<6.7%	<12.9%
2020	Zhang	31529619	5-6 ng/ml	5.0-6.0	serum	ctrls: 25.98 ± 2.73, T2D: 26.32 ± 3.41	ctrls: 54.62 ± 5.97; T2D: 56.40 ± 7.49	120, 54/66	Human ELISA kit; Wuhan EIAab Science Co. Ltd., Wuhan, China				
2020	Silistre	32003085	ctrls: 70.903 ± 17.49 ng/ml; overweight: 79.744 ± 29.54 ng/ml; obese: 106.293 ± 122.69 ng/ml	53.41-186.03	serum	ctrls: 19.745 (7.82), overweight: 24.32 (10.49), obese: 29.585 (20.56)	12.589 ± 2.42	158, 77/81	HumanAsprosin Elisa Kit, catalogue number: SG-15241 (SinogeneclonHang Zhou, China)	7.80 - 500 ng/ml	2.1 ng/ml	<8%	<10%
2020	Zhong	32090964	ctrls: 0.5 (1.13) ng/ml; gestational diabetes mellitus (GDM): 1.35 (0.92) ng/ml	0.5-1.35	plasma	ctrls: 25.9 ± 2.48, GDM: 25.01 ± 5.28	34.18 ± 3.24	80, 80/0	Human Asprosin ELISA Kit. Catalogue No: abx257694, Abbexa Ltd, Cambridge, UK		<0.938 ng/ml	<10%.	<8%
2020	Zhang	32458209	ctrls: 5.08 ± 1.31 ng/ml, non diabetic kidney disease (nDKD): 6.23 ± 0.87 ng/ml, DKD: 7.23 ± 0.94 ng/ml	3.77-8.17	serum		49.38 - 64.57	105, 52/53	Human ELISA kit, Wuhan EIAab Science Co. Ltd., China				
2020	Ke	32645536	ctrls: 1.54 ± 0.47 ng/ml, NAFLD: 2.27 ± 1.05 ng/ml	1.07-3.32	serum	ctrls: 22.40 ± 2.99, NAFLD: 25.24 ± 2.68	ctrls: 53.10 ± 12.64, NAFLD: 52.98 ± 14.88	93	Human Asprosin ELISA Kit. Catalogue No: abx257694, Abbexa Ltd, Cambridge, UK		<0.938 ng/ml	<10%.	<6%
2020	Naiemian	32714446	ctrls: 3.50 (1.85) ng/ml, T2D: 4.18 (4.4) ng/ml	1.65-8.58	serum	ctrls: 26.66 (3.01), T2D: 27 (3.27)	52-54	194, 94/100, ctrls: 97, T2DM: 97	Cat. No: CK-E91570; EASTBIOPHARM, China			<10%	<12%
2020	Ceylan	32741294	morning ctrl: 0.70 ±.17 ng/ml, OW/OO: 0 .97 ±.26 ng/ml; evening ctrl: 0.66 ±.12 ng/ml, OW/OO: 0.97 ±.26 ng/ml	0.53-1.17	serum	ctrls: 18.5–24.9, OW/OO: 25–29.9 or 30–39.9	30-45	20, 0/20	E-EL-H2266-Elabscience, Biotechnology	0.31 - 20 ng/ml		<10%	
2020	Wen	32894050	ctrls: 10-480 ng/ml, dilated cardiomyopathy: 50-220 ng/ml	10-220	serum	25.8-26.4	54-55	50, 7/43	capture antibody: mouse monoclonal anti-asprosin against: human profibrillin aa 2832–2871, detector: polyclonal goat anti-asprosin (human profibrillin aa 2737–2750) by Abnova				
2020	Deng	32894050	T2DM/Normoalbuminuria: 1.59(1.18–2.09) ng/ml, Microalb.: 2.10(1.60–2.90) ng/ml, Macroalb.: 2.37(1.63–3.57) ng/ml	1.18-3.57	serum	24.22 ± 3.45, 24.93 ± 3.43, 25.81 ± 2.84	60.15 ± 10.56, 62.96 ± 10.87, 63.40 ± 10.51	207, 44/63, 33/47, 10/10	Jiangsu, Feiya biological technology, Jiangsu, China; catalogue No. MM-1650H1		0.1 ng/ml	<10%	<15%
2021	Hu	32026376	ctrls: 1947.0 ± 2143.8 pg/mL; anorexia nervosa: 2514.8 ± 1957.2 pg/mL	0-4.47	plasma	ctrls: 19.0 ± 1.7, anorexia nervosa: 15.0 ± 2.0	ctrls: 18.7 ± 2.2, anorexia nervosa: 18.7 ± 2.2	46, 46/0	Asprosin (human) Matched Pair Detection Set (catalog number AG-46B-0011-KI01) pur-chased from Adipogen Ltd., San Diego, USA	0 - 5 ng/ml		<5%	<7%.
2021	Deniz	32608281	ctrls: 9 ± 2, PCOS: 28 ± 4 ng/ml	7.0-32.0	plasma	ctrls: 23.99 ± 4.2, PCOS: 24.77 ± 4.12	ctrls: 28.22 ± 2.6, PCOS: 27.14 ± 3.21	60, 0/60 (ctrls:30; PCOS: 30)	Human Asprosin ELISA Kit Bioassay TechnologyLaboratory, Catalogue no: E4095Hu Shanghai, CHIN	0.5 - 100 ng/ml	0.23 ng/ml	<8%	<10 %
2021	Du	32661697	ctrls: 1-18 ng/ml, cachexia, anorexia, post-operation: 1-25 ng/ml	1.0-25.0	plasma	22.7 (3.4)	<65: 83, >65: 37	120, 24/96	abx555449, AbbexaBiotech, UK				
2021	Leonard	33289860	oral contraceptive : 0.75 ± 0.38, non-OC: 1.00 ± 0.37 ng/ml	0.37-1.37	plasma	22.3 ± 2.8, 21.9 ± 1.9, 21.9 ± 2.7, 22.2 ± 2.4	24 ± 3, 27 ± 5, 24 ± 2, 25 ± 5	32, 32/0	Human Asprosin ELISA Kit. Catalogue No: abx257694, Abbexa Ltd, Cambridge, UK				
2021	Hoseini	33352518	30.8 (23.8 – 48.0) ng/ml	23.8 - 48.0	serum	23.83 ± 8.06	27.6+-5.6	759, 759/0	EASTBIOPHARM, China		0.34 ng/ ml	<10%	<12%
2021	Jiang	33766125	placebo ctrl: 33.43 (19.64, 49.94), SGLT2 inhibitor: 36.88 (24.81, 69.04)	19.64-69.04	serum	placebo: 25.64 ± 1.41, SGLT2 inhibitor: 26.60 ± 1.32	placebo: 59.3 ± 9.03, SGLT2 inhibitor: 58.32 ± 8.01	31, placebo: 8/2, SGLT2: 11/9	Eiaab Science INC. Wuhan, China (Catalogue Numbers, E15190h)	1.563 ng/ml to 100 ng/ml	0.938 ng/ml	<6.6%	<7.6%
2021	Hong	33747078	ctrls: 16.70 [12.87, 22.38] ng/mL, metabolic syndrome: 23.52 [16.70, 32.05] ng/mL	12.87-32.05	serum	ctrls: 23.28 ± 2.49, MetS: 25.52 ± 3.02	ctrls: 49.47 ± 9.16, MetS: 50.90 ± 9.45	295, ctrl: 84/78, MetS: 66/65	Human Asprosin ELISA Kit. Catalogue No: abx257694, Abbexa Ltd, Cambridge, UK				
2021	Corica	33662891	ctrls: 358.1 ± 74.1 pg/ml, obese: 331.9 ± 120.5 pg/ml	0.28-0.45	serum	obese vs lean	children	no information given	no information given				

Abbreviations

aa: amino acid, BMI: body mass index, ctrls: controls, CV: coefficient of variation, OO: obese overweight, OW: overweight, PCOS: polycystic ovary syndrome, MetS: metabolic syndrome, NAFLD: non-alcoholic fatty liver disease, T1(2)D: type 1 (2) diabetes, UAP: unstable angina pectoris

Supplementary Table S2: N-linked glycans expressed on recombinant asprosin expressed in HEK293 cells. MALDI mass spectrometric analysis of methylated glycans.

M+Na (experimental)	Structural asignment	Antennarity* -
1579.74	H5N2 (M5)	
1783.84	H6N2 (M6)	
1835.88	F1H3N4	2
1987.92	H7N2 (M7)	
2039.96	F1H4N4	2
2080.99	F1H3N5	2
2192.01	H8N2 (M8)	
2244.05	F1H5N4	2
2285.08	F1H4N5	3
2489.17	F1H5N5	3
2605.21	S1F1H5N4	2
2646.23	S1F1H4N5	3
2693.25	F1H6N5	3
2734.27	F1H5N6	4
2850.32	S1F1H5N5	3
2938.37	F1H6N6	4
3054.41	S1F1H6N5	3
3299.53	S1F1H6N6	4
3415.58	S2F1H6N5	3
3503.63	S1F1H7N6	4
3864.83	S2F1H7N6	4
4226.04	S3F1H7N6	4
4588.31	S4F1H7N6	4

*Antennarity is defined only for complex-type N-glycans. Structural asignments refer to monosaccharide compositions in terms of S, N-acetylneuraminic acid; F, fucose; H, hexose; and N, N-acetylhexosamine.

Supplementary Table S3: Peptides of de-N-glycosylated asprosin after double digestion with trypsin and V8.

1 ST**N**ETDASNI EDQSETEA**N**V SLASWDVEKT AIFAF**N**ISHV SNKVRILELL PALTTLTNHN RYLIESGNED GFFKINQKEG
81 ISYLHFTKKK PVAGTYSLQI SSTPLYKKKE LNQLEDKYDK DYLSGELGDN LKMKIQVLLH

Detected	m/z (mi)	m/z (av)	Modifications	Start	End	Missed Cleavages	Sequence	N-glycosylation site
**0**	450.1831	450.4278		1	4	0	(-)STNE(T)	**N3**
**1**	708.2683	708.6602		12	17	1	(E)DQSETE(A)	
**1**	1065.5728	1066.2526		80	88	0	(E)GISYLHFTK(K)	
**0**	1180.4964	1181.1618		1	11	1	(-)STNETDASNIE(D)	**N3**
**0**	1190.5688	1191.2906		18	28	0	(E)ANVSLASWDVE(K)	**N19**
**1**	1193.6677	1194.4278		80	89	1	(E)GISYLHFTKK(K)	
**1**	1194.6154	1195.3688		79	88	1	(K)EGISYLHFTK(K)	
**1**	**1318.6638**	1319.4658	**+1**	18	29	1	(E)A**N**VSLASWDVEK(T)	**N19**
**0**	**1420.6591**	1421.5125		16	28	1	(E)TEANVSLASWDVE(K)	**N19**
**1**	1463.8329	1464.7182		49	61	0	(E)LLPALTTLTNHNR(Y)	
**1**	**1548.7540**	1549.6877	**+1**	16	29	2	(E)TEA**N**VSLASWDVEK(T)	**N19**
**1**	**1548.8169**	1549.7806	**+1**	30	43	0	(K)TAIFAF**N**ISHVSNK(V)	**N36**
**1**	**1639.6566**	1640.5774	**+1**	1	15	2	(-)ST**N**ETDASNIEDQSE(T)	**N3**
**0**	**1676.9119**	1677.9558		29	43	1	(E)KTAIFAFNISHVSNK(V)	**N36**
**0**	**1803.9864**	1805.1027		30	45	1	(K)TAIFAFNISHVSNKVR(I)	**N36**
**1**	**1869.7468**	1870.7993	**+1**	1	17	3	(-)ST**N**ETDASNIEDQSETE(A)	**N3**
**0**	**1879.8192**	1880.9281		12	28	2	(E)DQSETEANVSLASWDVE(K)	**N19**
**0**	**1932.0814**	1933.2779		29	45	2	(E)KTAIFAFNISHVSNKVR(I)	**N36**
**0**	**2007.9142**	2009.1033		12	29	3	(E)DQSETEANVSLASWDVEK(T)	**N19**
**0**	**2159.1971**	2160.5398		30	48	2	(K)TAIFAFNISHVSNKVRILE(L)	**N36**
**0**	**2287.2921**	2288.7150		29	48	3	(E)KTAIFAFNISHVSNKVRILE(L)	**N36**
**0**	**2610.1326**	2611.6621		5	28	3	(E)TDASNIEDQSETEANVSLASWDVE(K)	**N19**
**0**	**2848.4628**	2850.2236		18	43	2	(E)ANVSLASWDVEKTAIFAFNISHVSNK(V)	**N19**, **N36**

Supplementary Table S4: Serum asprosin concentrations (mean ± SD) before (t0) and after (t1 – t4) treadmill exercise (n = 15 subjects).

**time points (min)**	**asprosin (ng/ml)**	**95 % confidence interval**	
		**lower limit**	**upper limit**
t0 (0)	11.8 ± 6.2	8.4	15.2
t1 (30)	13.7 ± 6.5 (*)	10.1	17.4
t2 (60)	13 ± 7.1	9.1	16.9
t3 (90)	13.1 ± 6.5	9.5	16.6
t4 (120)	13.3 ± 7.6	9.1	17.5

*P = 0.0385

Supplementary Table S5: Serum asprosin concentrations (mean ± SD before (t0) and after (t1 - t3) total hip replacement (THR) surgery (n = 14 patients).

**time points (d)**	**asprosin (ng/ml)**	**95% confidence interval**	
		**lower limit**	**upper limit**
t0 (0)	21.0 ± 9.0	15.8	26.2
t1 (7)	14.8 ± 6.5 (***)	11.1	18.6
t2 (90)	19.1 ± 8.1	14.4	23.7
t3 (365)	16.8 ± 7.5	12.5	21.2

***P = 0.0003

Supplementary Table S6: Serum COMP concentrations (mean ± SD) before (t0) and after (t1 - t3) total hip replacement (THR) surgery (n = 14 patients).

time points	COMP (ng/ml)	95% confidence interval	
		lower limit	upper limit
t0	707.0 ± 171.0	608.3	805.7
t1	467.1 ± 124.1 (**)	395.4	538.8
t2	783.7 ± 286.2	618.5	949.0
t3	737.1 ± 216.0	612.4	861.7

**P = 0.0056

Supplementary Table S7: Anthropometric measures of the two analyzed cohorts in this study.

cohort	f/m	age (years)	height (m)	weight (kg)	BMI (kg/m^2)
treadmill exercise	0/15	27.5 ± 3.1	1.8 ± 0.05	78 ± 7.7	23.9 ± 1.9
total hip replacement (THR) surgery	7/7	61.4 ± 10.5	1.74 ± 0.07	79.7 ± 16.4	26.2 ± 4.7

Original gels and western blots

Markers were purchased from Thermo Fisher Scientific:
1- PageRuler Prestained Protein Ladder, 10 to 180 kDa (#26616)
2- PageRuler Plus Prestained Protein Ladder, 10 to 250 kDa (#26620)
3- Spectra Multicolor High Range Protein Ladder, 40 to 300 kDa (#26625)

Blot/gel images were arranged and edited for figure preparation using Microsoft PowerPoint 2016

Figure	percentage (%) SDS-PAGE gel	running condition	method of analysis	used antibody
1B	12	reducing	Coomassie	-
1D	10	reducing	Coomassie	-
1F(left)	10	reducing	WB	pc-asp
1F(middle)	7.5	non-reducing	WB	fibrillin-1 (rF90)*
1F(right)	7.5	non-reducing	WB	CPTC-FBN1-3*
1G	10	reducing	WB	pc-asp
3B(left)	10	reducing	Coomassie	-
3B(right)	10	reducing	WB	mab anti-asprosin (Birdy-1)
3C	10	reducing	WB	mab anti-asprosin (Birdy-1)
4A	7.5	non-reducing	WB	fibrillin-1 (rF90)*
S5D	10	reducing	WB	pc-asp
S5F	10	reducing	Coomassie	-
S5I	10	reducing	WB	mab anti-asprosin (Birdy-1)

*non-reducing condition was chosen, since antibodies recognize folded epitopes.

Figure 1B

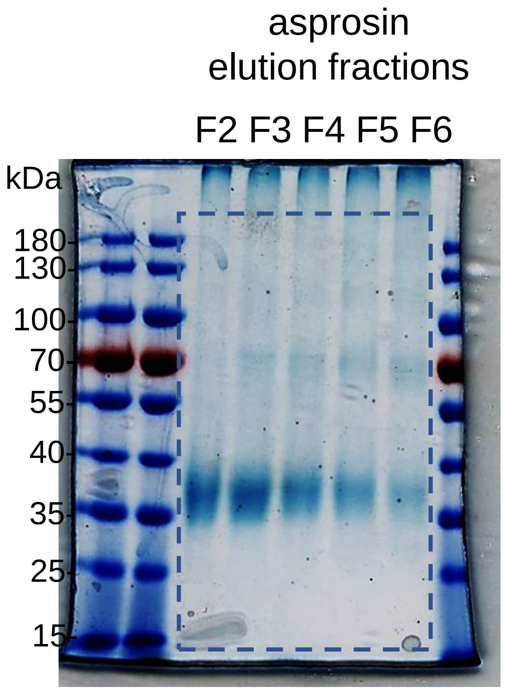

Figure1D

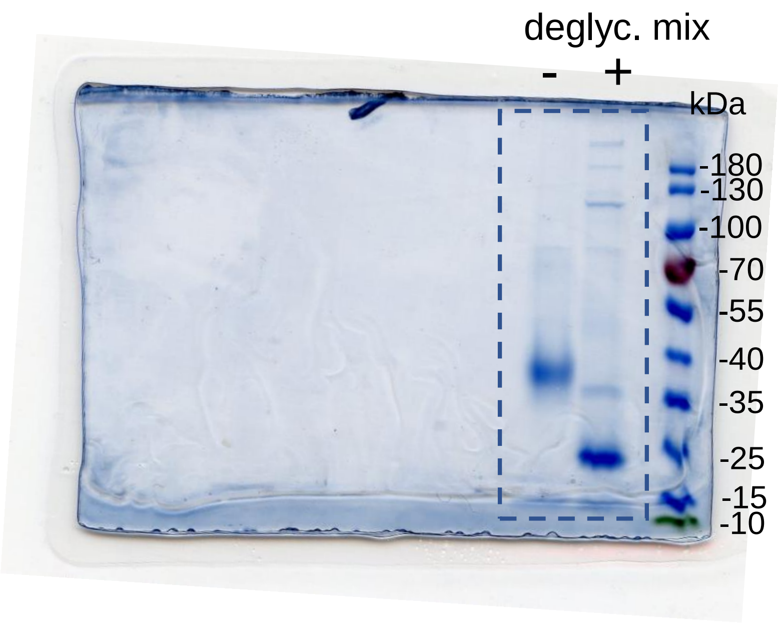

Figure 1F

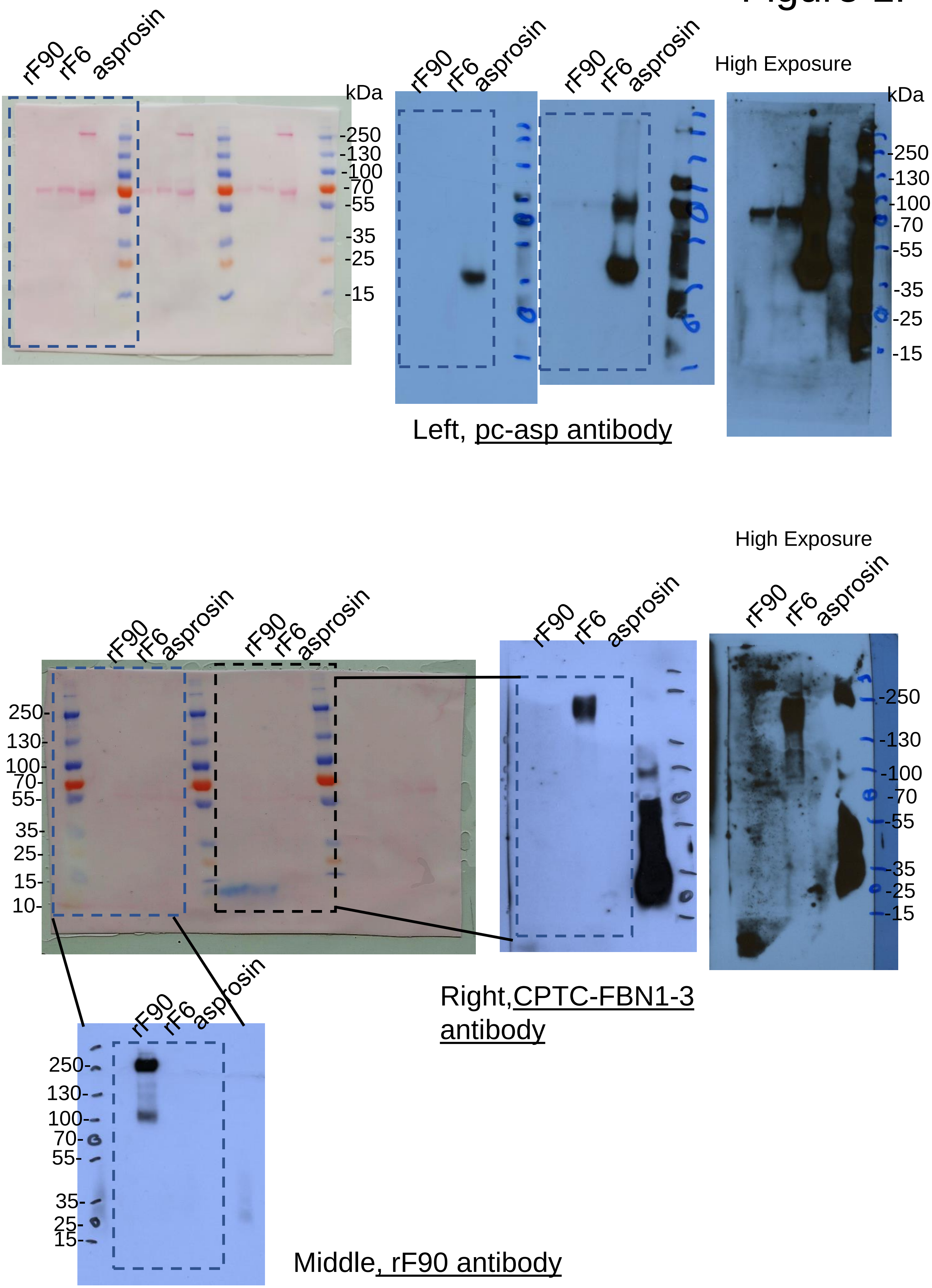
rF90
rF6
asprosin
kDa
-250
-130
-100
-70
-55
-35
-25
-15
High Exposure
Left, pc-asp antibody
250-
130-
100-
70-
55-
35-
25-
15-
10-
Right,CPTC-FBN1-3 antibody
Middle, rF90 antibody

Figure 1G

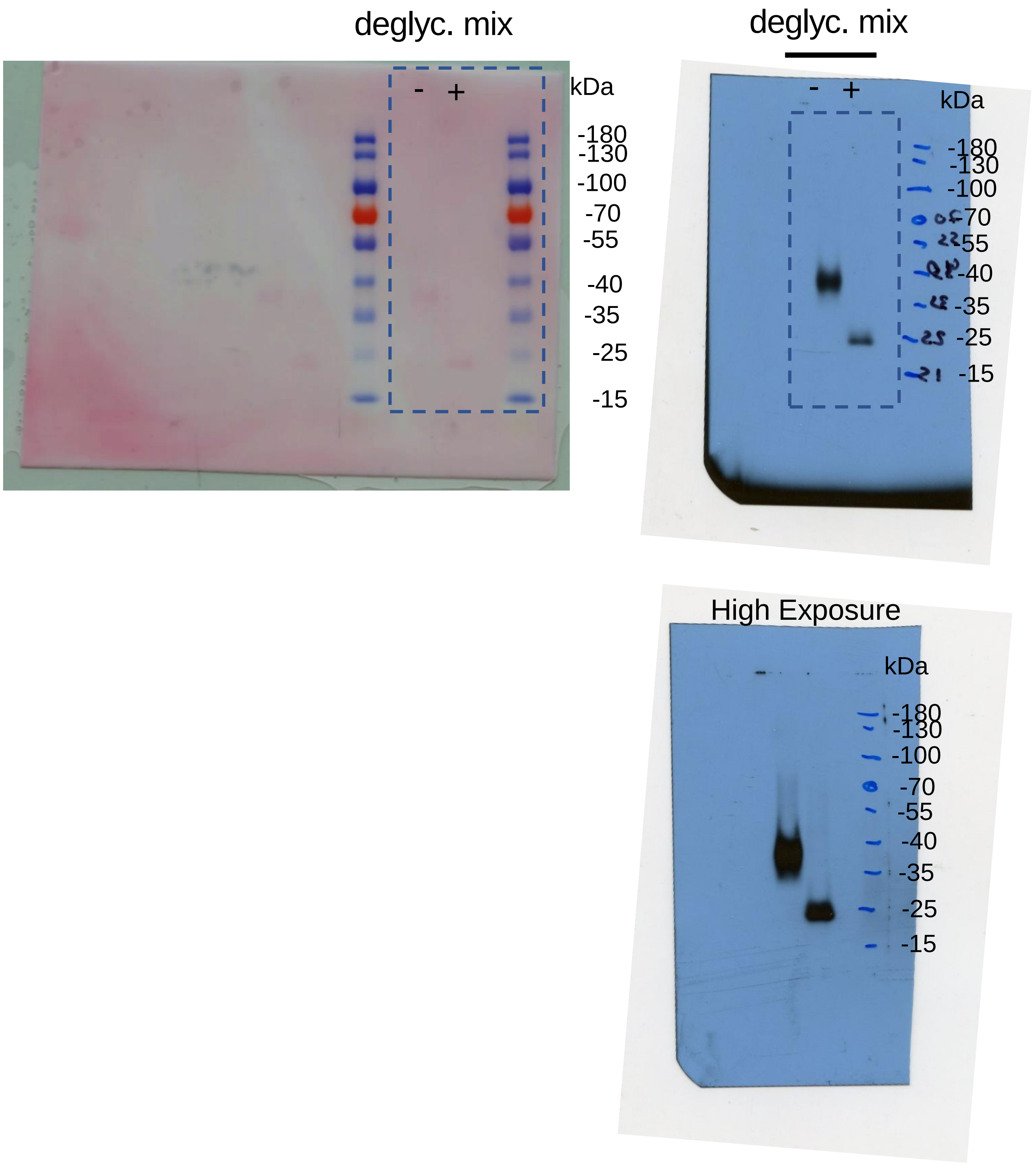
deglyc. mix
- +
kDa
-180
-130
-100
-70
-55
-40
-35
-25
-15
deglyc. mix
- +
kDa
-180
-130
-100
-70
-55
-40
-35
-25
-15
High Exposure
kDa
-180
-130
-100
-70
-55
-40
-35
-25
-15

Figure 3B

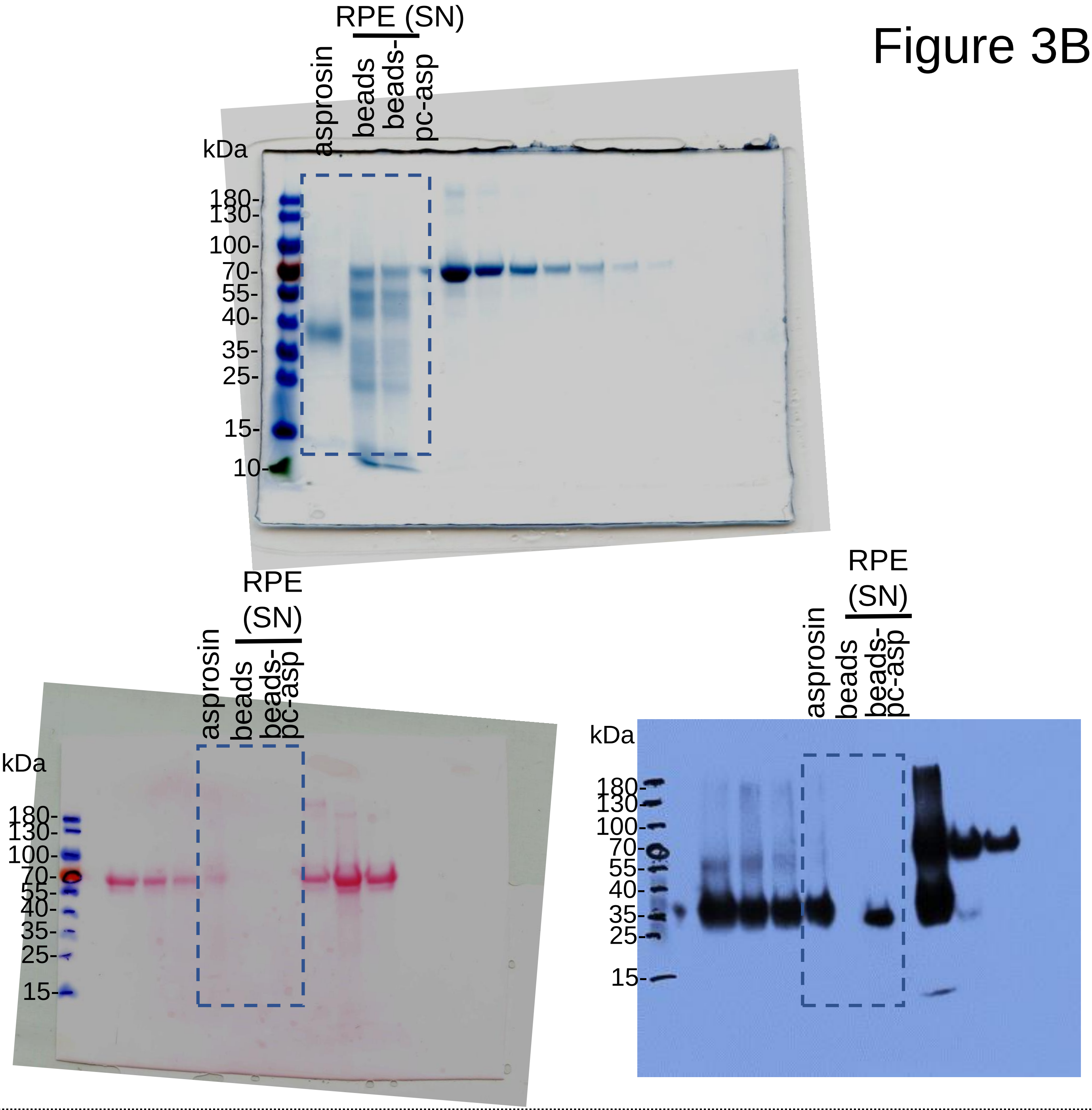

Figure 3C

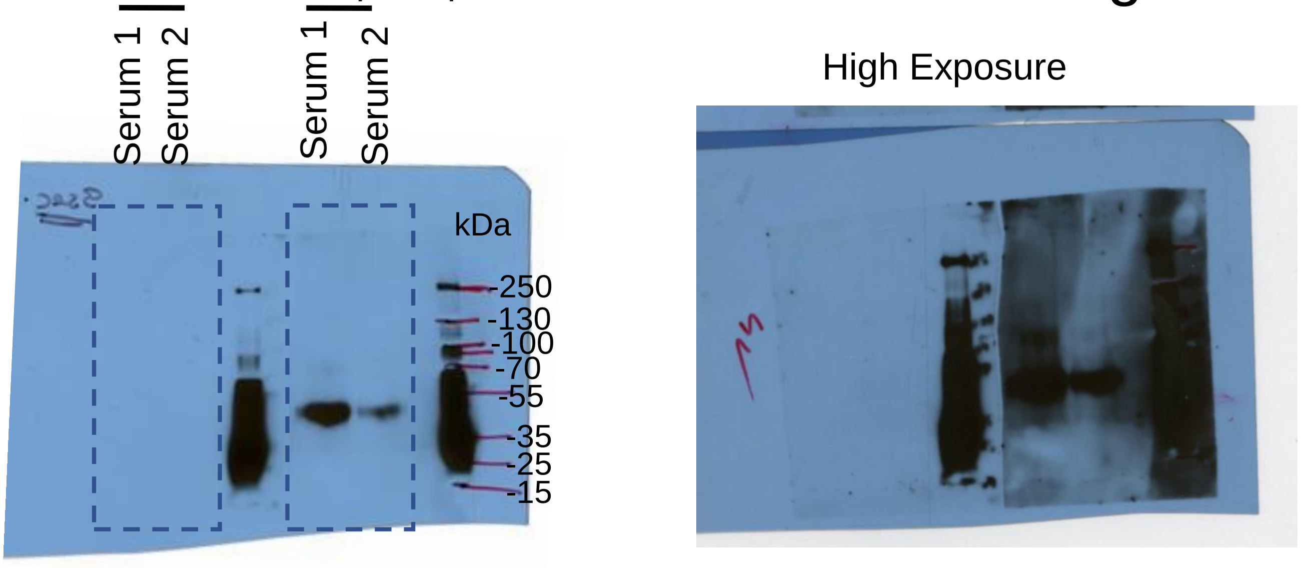

Figure 4A

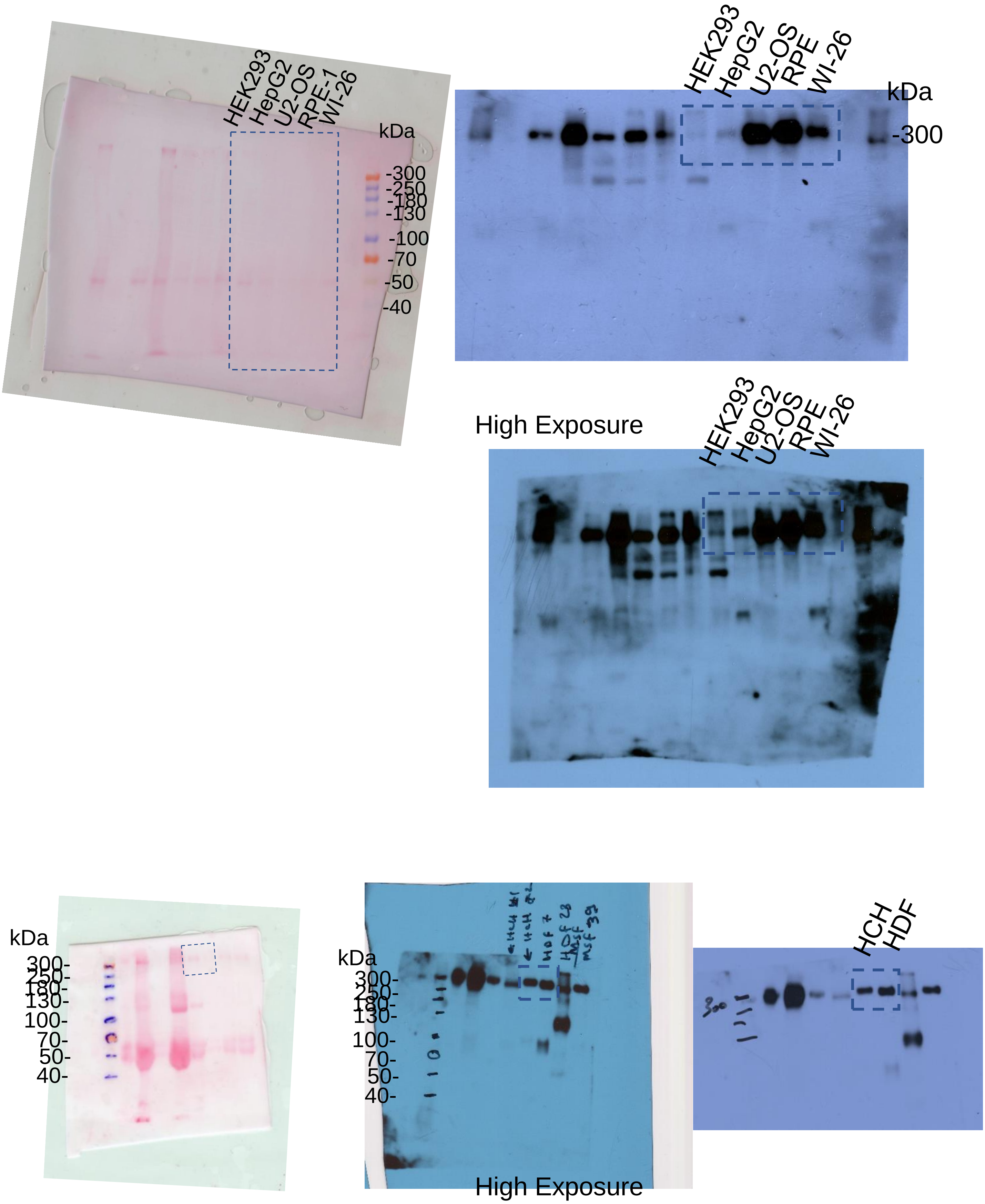

HEK293
HepG2
U2-OS
RPE-1
WI-26
kDa
-300
-250
-180
-130
-100
-70
-50
-40
HEK293
HepG2
U2-OS
RPE
WI-26
kDa
-300
High Exposure
HEK293
HepG2
U2-OS
RPE
WI-26
kDa
300-
250-
180-
130-
100-
70-
50-
40-
kDa
300-
250-
180-
130-
100-
70-
50-
40-
High Exposure
HCH
HDF

Figure S5D

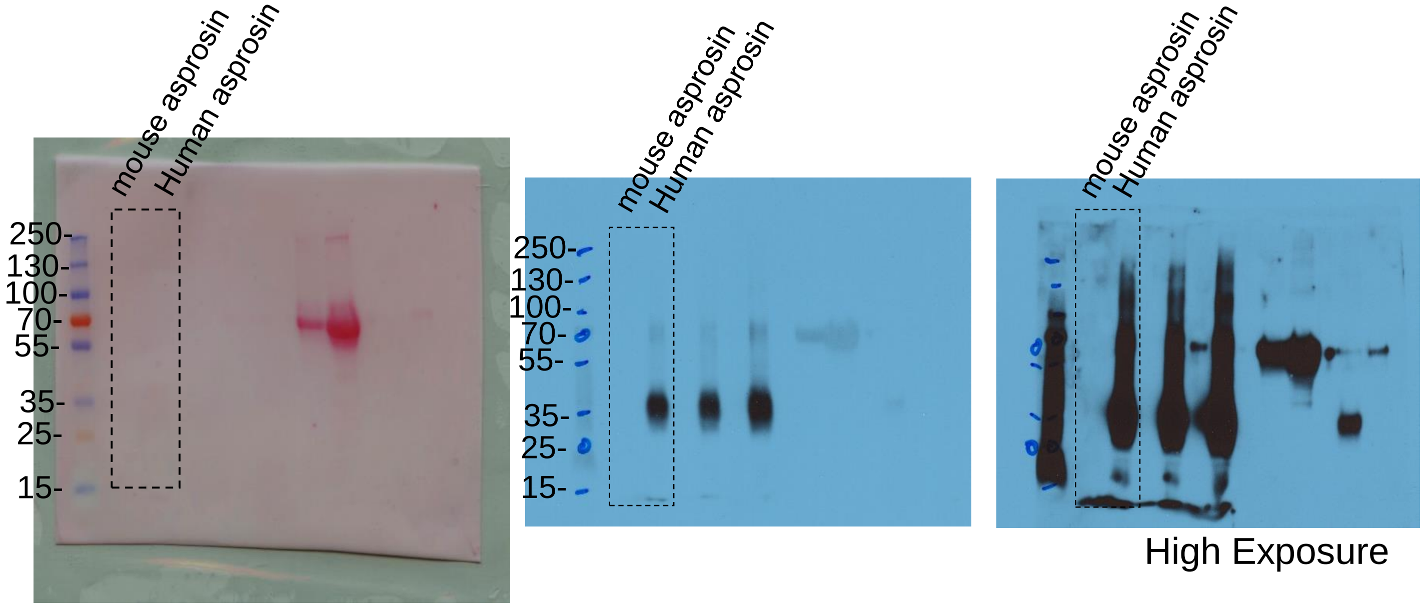

Figure S5F

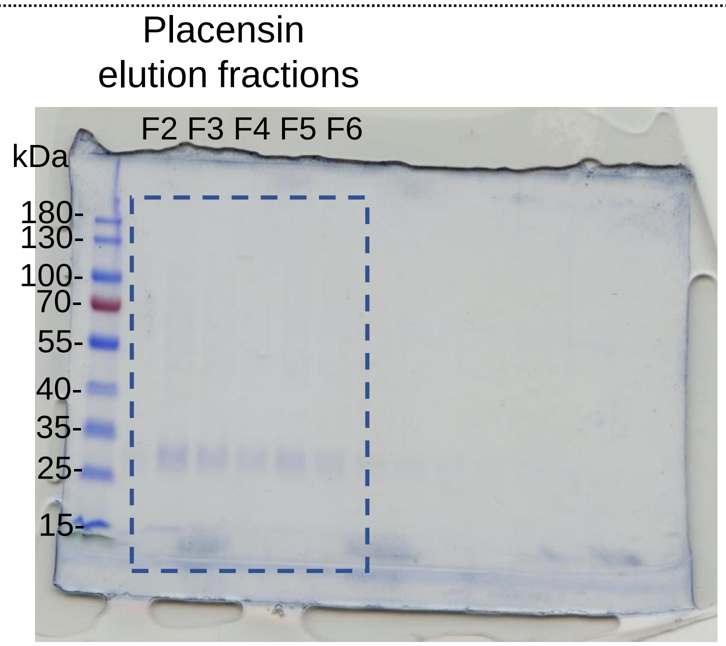

Figure S5I

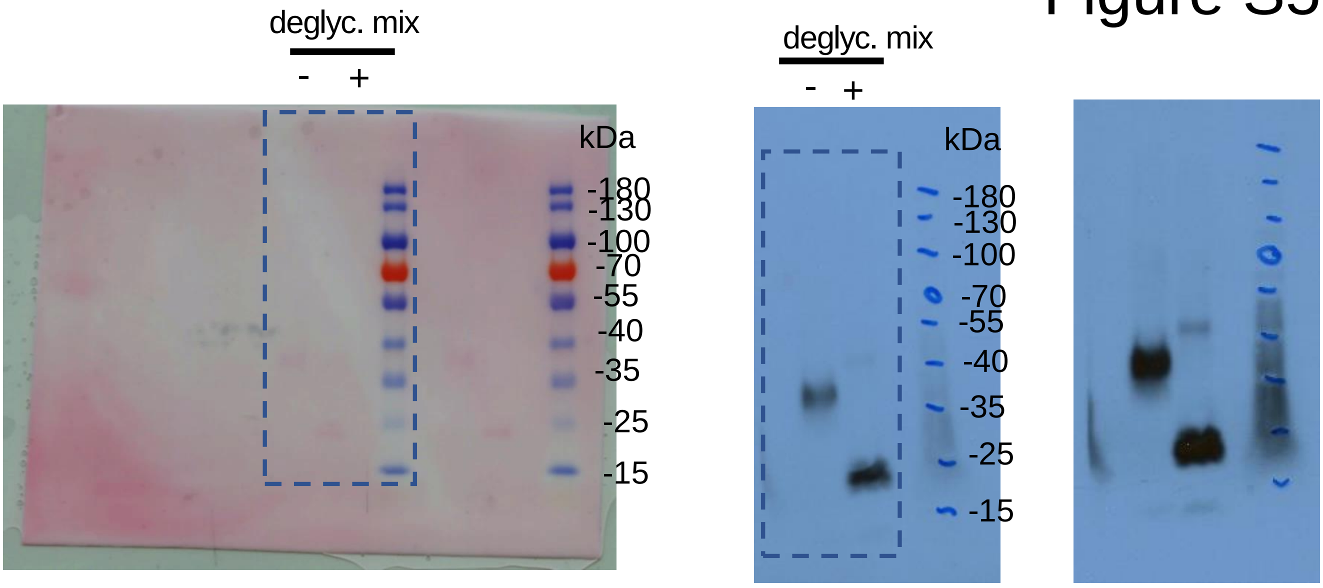

Supplement: Supplementary file 1 — Supplementary Information. [file 41598_2022_5060_MOESM1_ESM.pdf]
